# Integrated Analysis of Transcriptome and Metabolome Reveals Molecular Responses to Ammonia Stress in the Gills of *Litopenaeus vannamei* Under Low-Salinity Conditions

**DOI:** 10.3390/biology15080612

**Published:** 2026-04-13

**Authors:** Yutong Zhao, Yangyang Ding, Falin Zhou, Xiaojuan Hu, Qibin Yang, Yucheng Cao

**Affiliations:** 1 College of Fisheries and Life Sciences, Dalian Ocean University, Dalian 116023, China; yuton2025@163.com (Y.Z.);; 2 South China Sea Fisheries Research Institute, Chinese Academy of Fishery Sciences, Guangzhou 510300, China; dingyangyang93@163.com (Y.D.);; 3 Southern Marine Science and Engineering Guangdong Laboratory (Zhuhai), Zhuhai 519082, China; 4 Sanya Tropical Fisheries Research Institute, Sanya 572018, China

**Keywords:** ammonia nitrogen stress, low-salt adaptation, transcriptome, metabolomics, *L. vannamei*

## Abstract

High ammonia nitrogen stress significantly compromises the survival of *Litopenaeus vannamei* under low-salinity conditions. However, existing studies predominantly focus on ammonia nitrogen responses under single stressors or normal seawater salinity. The molecular regulatory mechanisms, metabolic remodeling patterns, and key pathway interactions in shrimp subjected to high ammonia nitrogen stress under low-salinity environment remain unclear. In this study, we employed integrated transcriptomic and metabolomic analyses to unveil the underlying molecular responses and metabolic biomarkers in the gills of *L. vannamei* to ammonia stress under low-salinity conditions. First, *L*. *vannamei* underwent low-salinity acclimation from 30‰ to 5‰ salinity and was then reared for one week to acclimate to the experimental environment. Subsequently, shrimp were treated with 42.32 mg/L ammonia nitrogen for a consecutive 96 h period. Integrated transcriptomic and metabolomic analyses elucidated the stress response patterns in the gills of *L. vannamei* under low-salinity ammonia nitrogen exposure. Specifically, 352, 802, and 140 differentially expressed genes (DEGs) were identified at 12 h, 48 h, and 96 h post-exposure, respectively. GO and KEGG enrichment analyses revealed that the significant DEGs were primarily enriched in six major pathways: autophagy, immune-related pathway, ABC transporter, fatty acid degradation and metabolism, metabolic pathway, and PPAR signaling pathway. Metabolomic profiling identified numerous differentially accumulated metabolites (DAMs) in both positive and negative ion modes, with significantly altered DAMs mainly consisting of organic acids and their derivatives, phospholipids, and other related metabolites. Key DAMs included taurine, guanosine, 1-palmitoyl-sn-glycero-3-phosphocholine, pseudouridine, and betaine. Integrative multi-omics analysis revealed that *L. vannamei* mediates stress responses by modulating five core pathways under low-salinity/high-ammonia-nitrogen dual stress: fatty acid degradation and metabolism (e.g., acyl-CoA dehydrogenase short chain (*Acads*), acetyl-CoA acetyltransferase 2 (*ACAT2*)), autophagy (e.g., autophagy-related protein 101-like (*atg101*)), immune regulation pathway (e.g., V-type proton ATPase subunit H-like (*VhaSFD*), actin-5C-like (*Act5C*)), metabolic pathway (e.g., molybdopterin synthase catalytic subunit-like (*Mocs2B*), cytochrome P450 2U1-like (*Cyp2b1*)), and ABC transporter (e.g., ATP-binding cassette sub-family D member 3-like (*ABCD3*), ATP-binding cassette sub-family B member 10 (*ABCB10*)). Through characterization of these core pathways, this study reveals the fundamental mechanisms by which *L. vannamei* responds to high ammonia nitrogen stress following low-salinity acclimation, providing a theoretical foundation for estuarine shrimp farming.

## Simple Summary

The farming of *L. vannamei* is widely carried out around the world, but high levels of ammonia in pond water often cause serious stress and even large-scale death in shrimp, especially under low-salinity conditions commonly found in coastal and estuarine areas. To better understand how shrimp respond to ammonia stress at low salinity, this study examined the changes in genes and small molecules in the gills of shrimp, which are key organs for breathing and environmental adaptation. We found that ammonia stress significantly disturbs energy supply, ion balance, and immune-related processes in shrimp gills. The results reveal the main biological ways that shrimp use to resist ammonia harm at low salinity. These findings can help farmers develop more effective methods to reduce stress damage, improve shrimp survival, and support healthier and more stable shrimp farming production.

## 1. Introduction

*Litopenaeus vannamei* (*L. vannamei*), commonly referred to as the Pacific white shrimp, is a decapod crustacean belonging to the family Penaeidae, natively distributed along the Pacific coast of Central and South America [1]. As a representative species in global shrimp aquaculture, it exhibits prominent advantages including rapid growth, high feed conversion efficiency, and strong environmental adaptability [2]. According to fisheries statistics, the production of *L. vannamei* has maintained steady growth in China. Meanwhile, estuarine low-salinity aquaculture has become a key development direction for the industry. In recent years, traditional marine shrimp farming has been restricted by coastal resource shortages, high disease risks, and environmental pressures. Estuarine low-salinity regions are widely distributed with stable water quality and low pathogen pressure. Benefiting from the strong low-salinity adaptability of *L. vannamei*, developing low-salinity aquaculture helps expand the culture scale and support the sustainable development of the shrimp industry [3,4].

For estuarine aquaculture, the dominant farming models include intensive recirculating aquaculture systems, closed-loop zero-water-exchange high-density farming, and earthen pond ecological farming [5,6,7]. In these systems, environmental factors profoundly affect the physiological status of *L. vannamei*, and ammonia nitrogen is one of the most important environmental stressors affecting shrimp health [8]. According to aquaculture water quality standards, the safe upper limit of ammonia nitrogen is 0.2 mg/L, but this threshold is often exceeded in practical culture [9]. High ammonia nitrogen directly damages gill tissue structure, impairs antioxidant systems and metabolic homeostasis, and suppresses immune function, making shrimp more vulnerable to pathogenic infection and causing high mortality and economic losses [10,11,12]. *L. vannamei* primarily relies on its intrinsic non-specific immunity to counteract environmental stress when subjected to adverse environmental conditions [13]. Thus, the expression of the shrimp’s immunity-related genes undergoes alterations upon exposure to ammonia nitrogen stress or salinity stress, which is reflected in the significant changes in the total antioxidant capacity (*T-AOC*), as well as the mRNA expression levels of heat shock protein 70 (*hsp70*) and thioredoxin (*TRX*) genes with the intensification of ammonia and salinity stress [14]. Furthermore, acute ammonia nitrogen exposure has been shown to increase the mRNA expression of kelch-like ECH-associated protein 1 (*keap1*) while downregulating nuclear factor erythroid 2-related factor 2 (*nrf2*) in the hepatopancreas of *Penaeus japonicus*, indicating a disruption of the antioxidant system [15]. Ammonia nitrogen stress also induces endoplasmic reticulum (ER) stress in the hepatopancreas of *L. vannamei*, as evidenced by the significant upregulation of marker genes such as immunoglobulin heavy chain-binding protein (*bip*), activating transcription factor 4 (*atf4*), and X-box binding protein 1 (*xbp1*) [16]. Moreover, such stress triggers apoptosis in the hepatopancreas and haemocytes of *L. vannamei*; for instance, studies under high-salinity conditions have confirmed that *lvmtbp* modulates the *p53-mdm2* axis and apoptotic pathways [17,18]. Beyond cellular damage, ammonia nitrogen impairs metabolic and osmoregulatory functions [19]. Transcriptomic analysis indicates that high ammonia nitrogen significantly upregulates genes associated with carbohydrate and lipid metabolism (e.g., hexokinase (*hk*) and medium-chain-specific acyl-CoA dehydrogenase (*acadm*)), and such upregulation indicates increased energy demand [20]. Conversely, ammonia nitrogen stress downregulates PPAR-α in gill tissue, reducing the activity of ion transport enzymes (e.g., Na^+^/K^+^-ATPase) and ultimately compromising osmoregulatory capacity [21]. Complementary metabolomic studies have further demonstrated that ammonia exposure disrupts amino acid, nucleotide, and lipid metabolism in shrimp [22].

Previous studies have mainly focused on ammonia nitrogen stress under high-salinity conditions (>25‰) or on small-sized *L. vannamei* (defined as individuals with body weight ≤ 10 g, while those > 10 g are classified as large-sized) [14,20,23,24]. In contrast, investigations concerning large-sized shrimp under low-salinity conditions remain limited. In commercial low-salinity aquaculture of acclimated *L. vannamei*, unexplained abnormalities, such as sudden mortality and muscle opacity, occur frequently [25,26,27]. The lack of targeted prevention and control strategies in aquaculture practices has caused substantial economic losses [25,26,27]. Therefore, elucidating the molecular mechanisms underlying the tolerance of *L. vannamei* adapted to low-salinity to high ammonia nitrogen stress is of great significance.

This study adopted an integrated transcriptomic and metabolomic approach to investigate the effects of ammonia nitrogen stress on the gill tissues of low-salinity-acclimated *L. vannamei*, thereby elucidating the stress response patterns and regulatory mechanisms underlying their adaptive processes. These findings establish a theoretical foundation for the optimal utilization of low-salinity aquaculture environments and provide novel insights into the molecular mechanisms governing the adaptation of *L. vannamei* to ammonia nitrogen stress under low-salinity conditions.

## 2. Materials and Methods

### 2.1. Experimental Shrimp

The experimental *L. vannamei* were sourced from the Shenzhen Experimental Base, South China Sea Fisheries Research Institute, Chinese Academy of Fishery Sciences. Prior to the formal experiment, the shrimp underwent a seven-day acclimation period in indoor rearing tanks (dimensions: 5.2 m (length) × 3 m (width) × 1 m (water depth)) at a stocking density of 100 individuals per cubic meter. During this adaptation period, the shrimp exhibited stable feeding behavior, with an average body length of (13.2 ± 0.5) cm and an average body weight of (14.6 ± 0.5) g. During the pre-culture period, water quality parameters were stably maintained at the following levels: temperature (30 ± 0.2) °C, pH 7.2–7.3, salinity (27 ± 1)‰, dissolved oxygen (DO) concentration ≥ 19.24 mg/L, ammonia nitrogen concentration ≤ 0.03 mg/L, and nitrite nitrogen concentration ≤ 0.08 mg/L.

### 2.2. Low-Salinity Acclimation and Stabilization

Prior to exposure to ammonia nitrogen stress, the shrimp were acclimated to the experimental low-salinity environment (5‰) for 7 days. During this period, the salinity was gradually reduced from 25‰ to 5‰ within 7 days, decreasing by 5‰ daily for the first 2 days and by 2‰ daily for the subsequent 5 days, followed by 7 days of stable acclimation at 5‰. This acclimation protocol was selected based on previous studies [28,29], which demonstrated that 7 days was sufficient for *L. vannamei* to fully adjust their osmoregulatory physiology to low-salinity conditions without inducing chronic stress. During both the acclimation and stabilization phases, water exchange was performed using drainage pumps and siphon hoses. Over the 7 days low-salinity acclimation period, the cumulative mortality of the shrimp was less than 3%, indicating that the shrimp successfully adapted to the low-salinity environment without severe physiological stress.

Following desalination, the average body length and body weight of *L. vannamei* were (14.0 ± 0.5) cm and (17.75 ± 0.5) g, respectively. After an additional 7 days stabilization period under low-salinity conditions, the average body length and body weight reached (14.6 ± 0.5) cm and (18.15 ± 0.5) g, respectively. During the entire experimental period, the shrimp maintained stable feeding behavior, with no clinical signs of disease or other physiological abnormalities observed.

With regard to water quality parameters, the pH, ammonia nitrogen, nitrite nitrogen, dissolved oxygen, salinity, and temperature of the aquaculture water are monitored daily to ensure that all environmental parameters remain within the range suitable for *L. vannamei.*

### 2.3. Acute Ammonia Nitrogen Stress

In this study, ammonium chloride (NH_4_Cl) was used and dissolved in aquaculture water to simulate a high-concentration ammonia nitrogen stress environment. Prior to the ammonia nitrogen stress treatment, the shrimp must be acclimated in the experimental tanks for 48 h to minimize handling stress. In the preliminary acute toxicity test, nine total ammonia nitrogen (TAN) concentrations (0, 5, 10, 15, 20, 30, 40, 50, and 60 mg/L) were set. Each concentration included three parallel tanks with 30 shrimp per tank (200 L water volume). Mortality was recorded every 3 h during the 96 h exposure period.

After 96 h of exposure, cumulative mortality was 3.3% at 0 mg/L, 10.0% at 5 mg/L, 16.7% at 10 mg/L, 23.3% at 15 mg/L, 29.0% at 20 mg/L, 38.0% at 30 mg/L, 47.0% at 40 mg/L, 62.0% at 50 mg/L, and 83.3% at 60 mg/L. The mortality of the control group was no more than 3.3%, and shrimp mortality increased gradually with elevated TAN concentration. Based on the mortality data, the 96 h LC_50_ of TAN was calculated as 42.32 mg/L using IBM SPSS Statistics 25, which was selected as the optimal concentration for acute ammonia nitrogen stress treatment. This concentration was chosen to mimic acute ammonia nitrogen spikes commonly observed in intensive low-salinity shrimp culture systems, where sudden elevations in ammonia often occur due to high feeding rates, organic matter accumulation, or temporary hypoxia. This concentration of TAN was used to simulate acute ammonia nitrogen spikes that frequently occur in intensive shrimp culture systems, especially under conditions of high organic loading or low dissolved oxygen. In the acute ammonia nitrogen stress treatment (42.32 mg/L TAN), the cumulative mortality rate at 96 h was consistent with the calculated LC_50_ value, and no unexpected mass mortality occurred in any treatment group, indicating that the stress intensity was appropriate for assessing physiological responses.

Low-salinity-acclimated *L. vannamei* (body length: 14.6 ± 0.5 cm; body weight: 18.15 ± 0.5 g, and healthy individuals with intact appendages, no visible disease) were randomly divided to two groups: the ammonia nitrogen stress group and the control group. Each subgroup contained 30 shrimp in a 200 L water volume, equivalent to 150 individuals per cubic meter, which is comparable to the density used in intensive shrimp farming practices. Specifically, six parallel experimental subgroups were established based on the LC_50_, whereas three parallel blank control subgroups were set up. Feeding was suspended throughout the stress period, and the shrimp were continuously exposed to the ammonia nitrogen stress condition for 96 h.

Tank specifications were as follows: diameter 100 cm × height 85 cm, with a total capacity of 500 L. Each tank was filled with 200 L of 5‰ salinity aquaculture water and continuously aerated for 24 h to ensure sufficient dissolved oxygen levels. Subsequently, healthy shrimp were introduced into each tank and allowed to acclimate for 48 h before ammonia nitrogen administration. On the following day, the pre-calculated amount of ammonium chloride was added to each experimental tank and vigorously mixed to ensure homogeneity. Subsequently, 100 mL water samples were collected from each tank, and the ammonia nitrogen concentration was determined in accordance with the national standard method (GB 17378.4-2007), to verify that the actual concentration was within the preset range. The initial ammonia nitrogen concentrations measured in all six culture tanks were within the range of (42.3233 ± 0.1) mg/L. Initial water quality parameters of each tank were measured and recorded as follows: salinity (5 ± 0.1) ‰, dissolved oxygen (>5 mg/L), pH (7.7 ± 0.1), nitrite nitrogen concentration ≤ 0.03 mg/L, water temperature (29 ± 0.5) °C, and ambient air temperature (30 ± 0.2) °C. Water quality parameters, including salinity, ammonia nitrogen, nitrite nitrogen, dissolved oxygen, pH, and water temperature, were measured at 0, 12, 48, and 96 h during the stress period, and all parameters remained relatively stable. Mortality was checked every 3 h.

During the formal ammonia nitrogen stress experiment, surviving shrimp were sampled at 12 h, 48 h, and 96 h post-stress. Specifically, after excluding shrimp exhibiting mortality or abnormal behavior during the experiment, six healthy shrimp were randomly selected: one individual from each of the six parallel experimental groups, and two individuals from each of the three control groups (CK). For the ammonia stress group, six biological replicates were used for both RNA-seq and metabolomics at each time point, with all samples analyzed individually. Each shrimp was randomly selected from a separate and independent tank to ensure robust biological replication.

Brief low-temperature anesthesia was applied to minimize shrimp stress before dissection. One side of the gill tissue was used for transcriptome analysis, and the other side was used for metabolome analysis. Each gill sample was weighed using an analytical balance, and the weight of both transcriptomic and metabolomic samples was greater than 250 mg per side. Using sterile dissecting instruments, the gill tissues of the selected shrimp were carefully dissected and immediately snap-frozen in liquid nitrogen for molecular analysis.

### 2.4. Transcriptomic Analysis

The collected gill tissue samples (80–100 mg per sample) were sent to Guangzhou OZ Bio Co., Ltd. (Guangzhou, Guangdong, China). for transcriptomic sequencing. Total RNA was extracted using the TRIzol reagent according to the manufacturer’s protocol. The TRIzol reagent was manufactured by Thermo Fisher Scientific (Waltham, MA, USA). Strict quality control (QC) was performed to assess RNA integrity, purity, and concentration via agarose gel electrophoresis, a NanoPhotometer spectrophotometer (NanoDrop 2000, Thermo Fisher Scientific, Waltham, MA, USA), and an Agilent 2100 Bioanalyzer (Agilent Technologies, Santa Clara, CA, USA); RNA samples with an RNA integrity number (RIN) ≥ 7.0 were qualified for library construction and sequencing. mRNA enrichment, fragmentation, and cDNA synthesis were performed to construct sequencing libraries, which were then subjected to paired-end (PE) sequencing on the Illumina platform to generate raw reads. Raw reads were quality-filtered to remove low-quality reads and adapter contamination, yielding high-quality clean reads. Clean reads were mapped to the reference genome of *L. vannamei* (GCF_042767895.1_ASM4276789v1) from the NCBI database. Inter-sample correlation and intra-group reproducibility were evaluated to ensure reliable replication. Significantly differentially expressed genes (DEGs) were identified with |log_2_ (fold change)| > 1 and adjusted *p* < 0.05, followed by GO and KEGG pathway enrichment analyses for functional annotation.

### 2.5. Metabolomics Analysis

The gill tissue samples were snap-frozen in liquid nitrogen after dissection and subsequently shipped to Guangzhou OZ Bio Co., Ltd. for non-targeted metabolomic analysis using liquid chromatography-mass spectrometry (LC-MS). Quality control (QC) samples were prepared by pooling equal volumes of all test samples for monitoring system stability. LC-MS data acquisition was performed in both positive ion mode (POS) and negative ion mode (NEG). Raw mass spectrometry data were processed using peak detection, filtration, and alignment to generate a standardized data matrix. Metabolites with more than 50% missing values were removed, and remaining missing values were imputed using the KNN (K-nearest neighbor) method. Data normalization was performed based on the total peak area to ensure comparability and reliability of metabolite identification and quantification across samples.

During data analysis, principal component analysis (PCA) was performed to evaluate the overall separation trends and intra-group reproducibility. Subsequently, differential metabolites were identified using Variable Importance in Projection (VIP), fold change (FC), and *p*-values. Finally, the screened differential metabolites were subjected to GO annotation and KEGG pathway enrichment analysis.

### 2.6. Integrated Analysis of Transcriptome and Metabolome

This study employed a multi-omics approach combining transcriptomics and metabolomics to elucidate the molecular response mechanisms of *L. vannamei* after high ammonia nitrogen stress at 5‰ salinity. Based on single-omics results, differentially expressed genes (DEGs) with |log_2_FC| ≥ 1 and *p* < 0.05 were identified, along with differentially metabolized compounds (DMs) meeting |log_2_FC| ≥ 1, *p* < 0.05 and VIP ≥ 1. DEGs underwent GO/KEGG enrichment analysis, while DMs were annotated via metabolic pathways in databases. Pearson correlation coefficients (|r| ≥ 0.5, *p* < 0.01) were then used to analyze gene–metabolite associations.

### 2.7. Quantitative Real-Time PCR

To minimize individual biological variation and obtain more stable and representative results, total RNA was extracted from pooled gill tissues of six shrimp collected at different time points (12 h, 48 h, 96 h) after exposure to high ammonia nitrogen stress under low-salinity conditions. Concurrently, total RNA was extracted from six pooled individuals of the control group (CK) that had not been exposed to high ammonia nitrogen stress at each corresponding time point. Ten significantly differentially expressed genes were randomly selected from the transcriptomic analysis for quantitative real-time PCR validation. These genes were randomly chosen from each key signaling pathway involved in this study, ensuring they were closely related to our research hypothesis and could represent the main biological processes.

Quantitative real-time polymerase chain reaction was performed using a CG-02 Fluorescence Quantitative PCR Instrument (Heal Force, China) and SYBR Green Master Mix (Accurate Biology, China). Three technical replicates were set for each reaction to ensure the reproducibility of quantification.

The specific primer sequences used for qPCR are listed in Table 1. All primers were designed using NCBI Primer-BLAST, and their amplification specificity was verified by melting curve analysis. All melting curves showed a single peak, confirming that the primers specifically amplified the target gene fragments without non-specific amplification or primer dimer formation.

### 2.8. Statistical Analysis

To account for tank-level variability, all biological replicates were obtained from independent tanks for both the ammonia stress treatment and control groups at each sampling point. For the treatment group, only one shrimp was collected from each independent tank; for the control group, two shrimp were collected from each independent tank. Environmental parameters (salinity, DO, pH, ammonia nitrogen, nitrite, temperature) were closely monitored and maintained consistent across all tanks throughout the experiment to minimize tank effects. All statistical analyses were conducted based on these independent biological replicates derived from separate tanks.

Bioinformatics analyses were performed using a suite of professional software and packages. For transcriptome data, raw reads were filtered using fastp; ribosomal RNA was removed using bowtie2; clean reads were mapped to the reference genome using HISAT2; and transcript assembly and quantification were performed using StringTie and RSEM. Differential gene expression was analyzed using the DESeq2 and edgeR packages in R software (Version 1.0.12, R Foundation for Statistical Computing, Vienna, Austria). Genes with FDR < 0.05 and |log_2_ (fold change)| > 1 were defined as significant DEGs. For metabolome data, raw MS data were converted using ProteoWizard (MSConvert, ProteoWizard Project, Palo Alto, CA, USA), processed using XCMS (Scripps Center for Metabolomics, La Jolla, CA, USA) and CAMERA (Bioconductor, Cambridge, MA, USA) for peak extraction, alignment, and annotation. Multivariate analyses (PCA, PLS-DA, OPLS-DA) were performed using the gmodels and ropls packages in R software. Metabolites with VIP ≥ 1 in the OPLS-DA model and *p* < 0.05 in Student’s *t*-test were considered significant. PCA was used to visualize overall differences and intragroup variability. Pearson correlation analysis was conducted using R software to evaluate repeatability of biological replicates. Hierarchical clustering analysis was performed using the pheatmap package in R.GO (Version 1.0.12, R Foundation for Statistical Computing, Vienna, Austria) and KEGG enrichment analyses were performed using the hypergeometric test, with corrected *p*-value (Q-value) ≤ 0.05 as the threshold. All statistical tests were two-sided, and *p* < 0.05 was considered statistically significant. For qPCR analysis, relative gene expression levels were calculated using the 2^−(ΔΔCt)^ method with β-actin as the internal reference gene. Statistical analysis and graphing were performed using Microsoft Excel and GraphPad Prism 10.1.2 software (GraphPad Software, LLC, San Diego, CA, USA). Prior to the two-tailed *t*-test comparing fold change values between qRT-PCR and RNA-seq, the Shapiro–Wilk test was used to verify normality, and Levene’s test was used to verify homogeneity of variance. The results confirmed that the data satisfied the assumptions of normality (*p* > 0.05) and homogeneity of variance (*p* > 0.05), validating the use of Student’s *t*-test.

## 3. Results

### 3.1. Transcriptome Analysis Quality Assessment

To systematically characterize the differential gene expression profiles in the gill tissues of *L. vannamei* subjected to high ammonia nitrogen stress following low-salinity acclimation, RNA-sequencing (RNA-seq) technology was employed on the Illumina platform in this study. Transcriptome sequencing of 36 gill tissue samples generated a total of 159 gigabytes (GB) of valid sequencing data. These 36 gill tissue samples were randomly collected from individual *L. vannamei*, with sampling conducted at three time points (12 h, 48 h, 96 h) for both the control group (designated as 12h-CK, 48h-CK, and 96h-CK) and the experimental treatment group (12 h, 48 h, 96 h), respectively. These 36 gill tissue samples were randomly selected from healthy individuals in each group at each time point before RNA extraction, without any selection based on RNA quality. Only samples with qualified RNA concentration, purity, and integrity were used for transcriptome sequencing.

Quality assessment was performed for all 36 transcriptome samples. These samples comprised six groups (three time points for control and three for treatment), with six biological replicates per group. Table 2 summarizes the average values of sequencing quality parameters (RawData, CleanData, LowQuality, GC, Q20, Q30, Total_Mapped, etc.) for each group. The sequencing quality was highly consistent among biological replicates within each group, with an average GC content of 42.74% and an average Q30 base percentage of 97.68%, verifying the high quality and good repeatability of the sequencing data (Table 2).

Furthermore, Pearson correlation principal component analysis (Figure S1A) and coefficient heatmap analysis (Figure S1B) revealed tight intra-group clustering, excellent sample reproducibility, and strong inter-sample correlations within each group, whereas distinct inter-group differences and clear separation were observed between the experimental and control groups. These results confirm the rationality of biological replicate design and the high reliability of the sequencing data.

### 3.2. Analysis of All Differentially Expressed Genes

**Figure 1:**
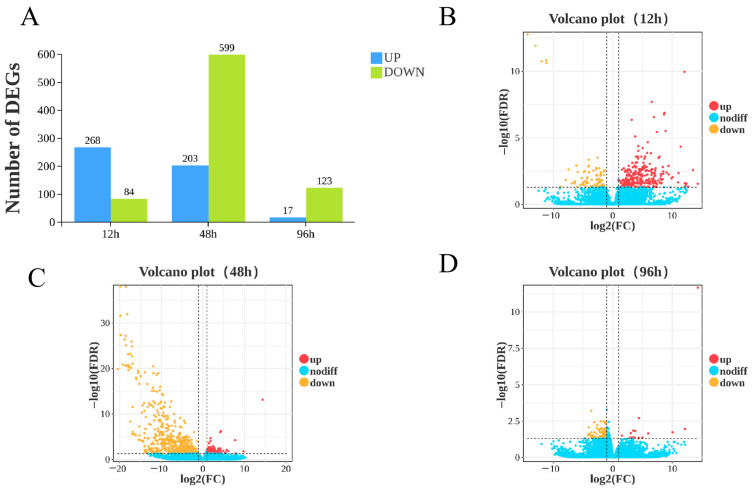
Cluster analysis of differentially expressed genes of the transcriptome. (**A**) Bar plots depict the counts of upregulated and downregulated DEGs at each sampling time point (12 h, 48 h, 96 h), with blue bars representing upregulated DEGs and green bars denoting downregulated DEGs. The number of DEGs at each stress time point was normalized against the control group. (**B**) Volcano plot illustrating the distribution of DEGs at 12 h; (**C**) volcano plot illustrating the distribution of DEGs at 48 h; (**D**) volcano plot illustrating the distribution of DEGs at 96 h. In all volcano plots, “up” indicates genes significantly upregulated at the corresponding time point, “down” indicates genes significantly downregulated at the corresponding time point, and “nodiff” indicates non-differentially expressed genes. DEG screening was performed under the criteria of FDR < 0.05 and |log_2_FC| > 1.

To comprehensively analyze the differences and dynamic changes in gill tissue gene expression between the control and experimental treatment groups (12 h, 48 h, 96 h) across the different time points, data visualization of the gene expression profiles was first performed based on transcripts per million (TPM) normalized values and volcano plots. Gene annotation and analysis based on the transcriptome sequencing data identified a total of 25,582 genes, including 1515 newly annotated genes. Using TPM as the gene expression quantification metric (Figure S2), a total of 25,596 expressed genes were detected. In gill tissue, following high ammonia nitrogen stress (stress concentration: 42.32 mg/L) under low-salinity conditions, 268, 203, and 17 genes were significantly upregulated at 12 h, 48 h, and 96 h, respectively (FDR < 0.05; |log_2_FC| > 1), while 84, 599, and 123 genes were significantly downregulated (FDR < 0.05; |log_2_FC| > 1) at 12 h, 48 h, and 96 h, respectively (Figure 1A).

Concurrently, volcano plot visualization analysis revealed that 12 h and 48 h post-stress represent key time nodes for significant gene expression changes (Figure 1B–D). These results indicate that the effects of high ammonia nitrogen stress on gill tissue gene expression in *L. vannamei* after low-salinity acclimation exhibit clear time-dependent patterns. Among all stress groups, the 48 h stress treatment group showed the most pronounced response, with the number of DEGs reaching a peak during this period and the overall gene expression trending downward.

### 3.3. GO Enrichment Analysis of DEGs

To identify functional candidate genes, the differentially expressed genes were subsequently subjected to functional annotation and characterization via GO functional enrichment analysis.

**Figure 2:**
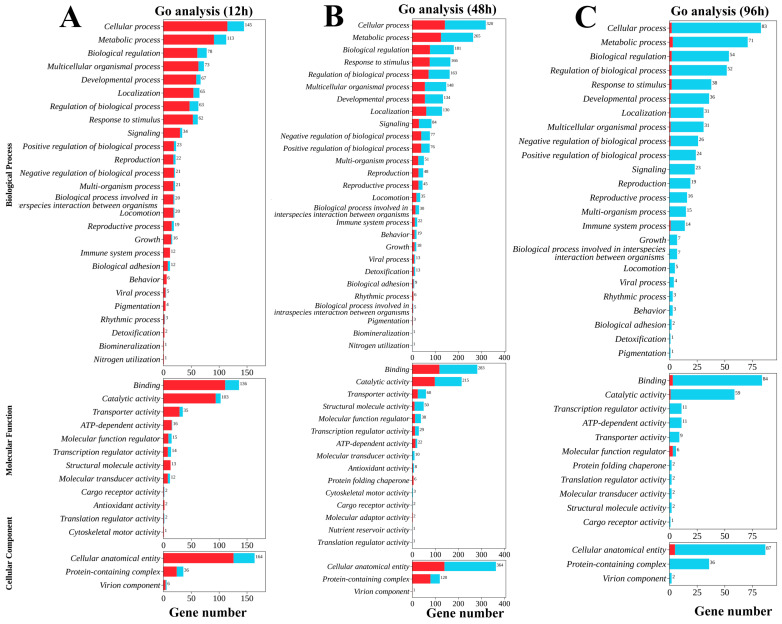
GO enrichment analysis of the transcriptome. (**A**–**C**) depict the results of GO enrichment analysis for *L. vannamei* under high ammonia nitrogen stress at 12 h, 48 h, and 96 h under low-salinity conditions. All enrichment results were classified into three core functional ontologies of the GO database: biological process (BP), cellular component (CC) and molecular function (MF). The *y*-axis of the plots represents the respective subcategories under these three core functional ontologies. In the figures, blue bar refers to the number of upregulated genes in the corresponding functional subcategory at the given stress time point, while red bar represents the number of downregulated genes in the relevant functional subcategory at the specified stress time point. The numbers displayed on these columns represent the total number of significantly differentially expressed genes within the respective GO analysis subcategory.

GO functional enrichment analysis of the significantly differentially expressed genes (Figure 2) showed that these DEGs were primarily categorized into the three main Gene Ontology (GO) categories: biological processes (BP), cellular components (CC), and molecular functions (MF). In the gill tissues of *L. vannamei* subjected to high ammonia nitrogen stress under low-salinity conditions, the subcategories with the highest DEG abundances at 12 h, 48 h, and 96 h post-stress were as follows: “cellular process” (145, 320, and 83 genes, respectively) under BP; “binding” (136, 283, and 84 genes, respectively) under MF, and “cellular anatomical entity” (164, 364, and 87 genes, respectively) under CC.

Notably, “metabolic processes” and “immune system processes” were significantly enriched in gill tissues of *L. vannamei* under the experimental conditions. Concurrently, following low-salinity acclimation, signal-transduction-related pathways associated with biological regulation, including “Signaling” and “transporter activity”, were also significantly enriched in the gill tissue in response to high ammonia nitrogen stress.

### 3.4. KEGG Enrichment Analysis of DEGs

To delineate the biological pathways perturbed in the gill tissues of *L. vannamei*, under the combined stress of low-salinity and high ammonia nitrogen, the DEGs identified in this study were mapped to the reference pathways within the KEGG database. With a significance threshold set at *p* < 0.05, KEGG enrichment analysis was performed to screen and identify the top 20 enriched pathways at each sampling time point.

**Figure 3:**
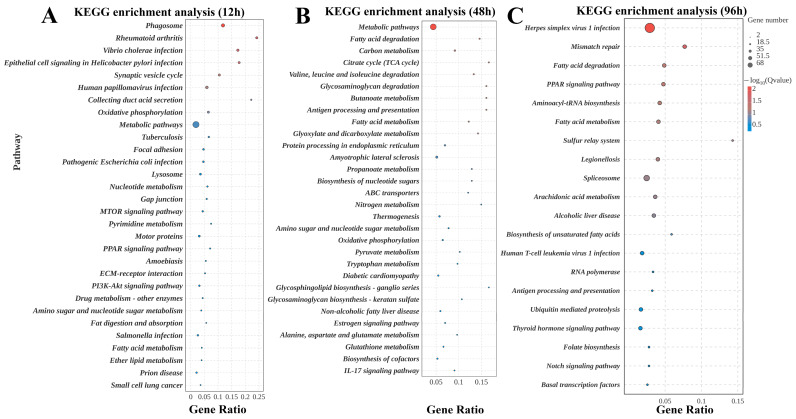
Transcriptomic KEGG Pathway Enrichment Analysis. (**A**–**C**) illustrate the KEGG pathway enrichment profiles at 12 h, 48 h, and 96 h post-exposure to high ammonia nitrogen stress following low-salinity acclimation. Pathways were screened under the criterion of *p* < 0.05, and the top 20 pathways with the lowest Q-values were selected for graphical presentation. The *y*-axis denotes the annotated KEGG pathways, while the *x*-axis represents the enrichment factor-defined as the ratio of the number of DEGs in a specific pathway to the total number of annotated genes in that pathway. Bubble size corresponds to the relative number of DEGs enriched in each pathway, and a progressively deeper red color indicates a smaller Q-value (higher enrichment significance).

After 12 h of exposure to high ammonia nitrogen stress under low-salinity acclimation conditions, the DEGs in gill tissues of *L. vannamei* were predominantly enriched in several key biological pathways, including the phagosome pathway, peroxisome proliferator-activated receptor (PPAR) signaling pathway, synaptic vesicle cycle, nucleotide metabolism pathway and pyrimidine metabolism pathway (Figure 3A). After 48 h of high ammonia nitrogen stress post low-salinity acclimation, metabolic pathways exhibited the highest enrichment of DEGs in the gill tissues of *L. vannamei*. This core pathway contains interconnected physiological and metabolic modules: (1) energy metabolism-associated sub-pathways (e.g., fatty acid metabolism, valine–leucine–isoleucine catabolism, tryptophan metabolism). (2) cellular structure and function maintenance pathways (e.g., glycosylation modification, lipid signaling). Furthermore, three additional pathways (e.g., ATP-binding cassette (ABC) transporter, glycosaminoglycan degradation, and pyruvate metabolism pathways) also reached the preset statistical significance threshold for enrichment (Figure 3B). After 96 h of stress, transcriptome analysis revealed that DEGs in gill tissues were primarily enriched in three key pathways: mismatch repair pathway, PPAR signaling, and fatty acid metabolism (Figure 3C).

Collectively, the results demonstrated that the gill tissues of *L. vannamei* exposed to high ammonia nitrogen stress under low-salinity conditions were predominantly perturbed in six key biological pathways, namely fatty acid degradation and metabolism pathway, autophagy pathway, immune response-related pathway, PPAR signaling pathway, metabolic pathway, and ABC transporter (Table 3). Furthermore, the metabolic pathway was found to harbor the largest number of differentially expressed genes (68 genes) in the gill tissues among all identified pathways.

### 3.5. qRT-PCR Validation of Representative DEGs

**Figure 4:**
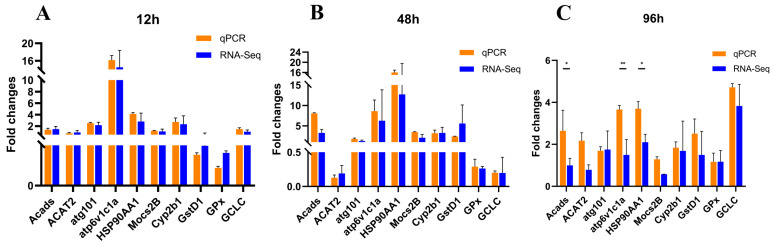
Validation of consistency between quantitative real-time PCR (qRT-PCR) and RNA sequencing (RNA-seq) results. (**A**–**C**) display the comparative results of fold change values of DEGs detected by qRT-PCR and RNA-seq at 12 h, 48 h, and 96 h post-stress, respectively. Orange bars represent qRT-PCR validation results: the *x*-axis shows 10 representative DEGs randomly selected from Table 3; the *y*-axis quantifies the relative expression fold change of each gene in the treatment group relative to the control group, calculated from Ct values using the 2^-(ΔΔCt)^ method. Blue bars represent RNA-seq detection results: the *x*-axis is consistent with that of qRT-PCR, i.e., the 10 representative DEGs mentioned above; the *y*-axis represents the fold change of the treatment group relative to the control group, computed based on the TPM expression values from RNA-seq analysis. A two-tailed *t*-test was performed on the fold change values of qRT-PCR and RNA-seq for the same gene at the same stress time point to verify the consistency of the two detection techniques. Significance levels are annotated as follows: *p* < 0.01 denoted by “**”; *p* < 0.05 denoted by “*”.

To verify the reliability of the transcriptomic sequencing (RNA-Seq) data generated in this study, gill tissue samples were randomly selected from the experimental and control groups at each stress time point for validation via real-time quantitative polymerase chain reaction (qRT-PCR). β-actin was used as the housekeeping gene for gene expression normalization. The fold change in gene expression levels detected by qRT-PCR was highly consistent with the expression profiles obtained from RNA-Seq, thus validating the accuracy and reliability of the present transcriptomic dataset (Figure 4 and Figure S3).

### 3.6. Basic Information of Metabolites

This study utilized LC-MS to perform non-targeted metabolomic profiling of gill tissues from *L. vannamei* Metabolite identification revealed a total of 22,131 metabolites detected across all experimental samples, with 10,369 metabolites identified in the positive ion detection mode (POS) and 11,762 in the negative ion detection mode (NEG), respectively (Table 4).

To further evaluate the reproducibility of the metabolomic dataset, a sample correlation heatmap was constructed based on Pearson correlation coefficients (Figure S4). Each grid in the heatmap represents the correlation coefficient between two corresponding samples. The coefficient value is positively correlated with color intensity, where higher coefficients correspond to darker color, indicating a higher degree of similarity in metabolic profiles between samples. The analysis showed that both the POS and NEG exhibited high intra-group correlation coefficients ranging from 0.8 to 0.9, whereas inter-group correlation coefficients varied from 0.5 to 0.6, with a minimum value of 0.2. These findings collectively demonstrate the excellent reproducibility of the metabolomics data and the pronounced metabolic differences between the experimental and control groups in this study.

### 3.7. Holistic Analysis of DAM Number Variations

To systematically characterize the differential metabolite profiles in the gill tissues of *L. vannamei* exposed to 42.32 mg/L ammonia nitrogen stress under low-salinity acclimation conditions, paired comparisons were performed between the stress samples and their corresponding control groups at 12 h, 48 h, and 96 h post-stress exposure. This study employed an integrated multivariate statistical analysis strategy combining orthogonal partial least squares discriminant analysis (OPLS-DA) and univariate *t*-tests to identify differentially accumulated metabolites (DAMs) across distinct control groups. The screening thresholds were set as follows: VIP ≥ 1 derived from the OPLS-DA model and statistical significance at *p* < 0.05 based on the *t*-test.

**Figure 5:**
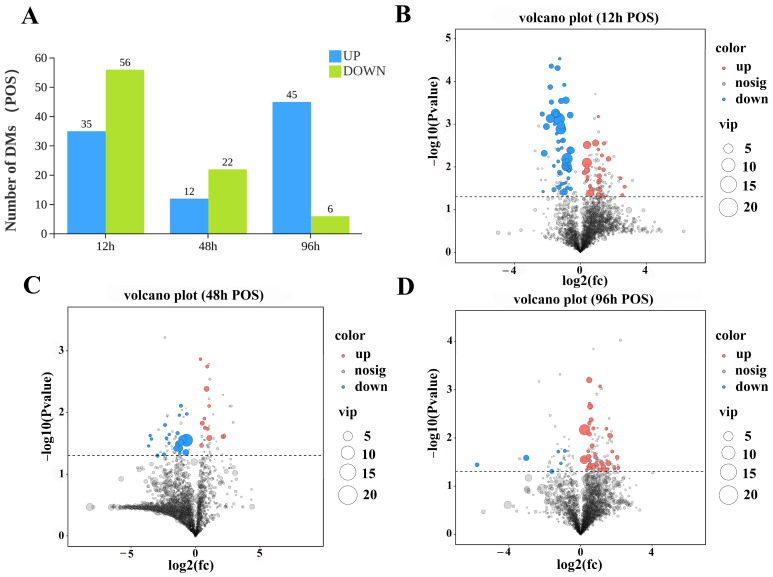
Metabolomics cluster analysis of significantly differentiated metabolites in POS. (**A**) Bar chart showing the number of significantly up- and downregulated metabolites at 12 h, 48 h, and 96 h (blue: upregulated; green: downregulated), normalized to the control group. (**B**–**D**) Volcano plots showing the distribution of significantly differentiated metabolites at 12 h, 48 h, and 96 h, respectively. Significantly differentiated metabolites were screened with FDR < 0.05 and |log_2_FC| > 1. *x*-axis: log-transformed fold change between groups; *y*-axis: −log10 *P* of group differences. Metabolites closer to the two ends of the *x*-axis have greater differential expression; different colors indicate up- or downregulated metabolites according to the threshold, and grey points indicate non-differentiated metabolites.

**Figure 6:**
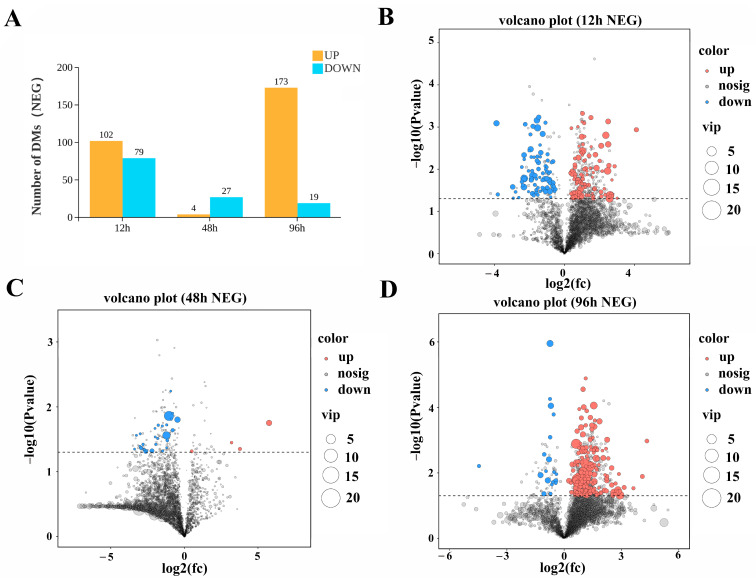
Metabolomics cluster analysis of significantly differentiated metabolites in NEG. (**A**) Bar chart showing the number of significantly upregulated and downregulated metabolites at 12 h, 48 h, and 96 h (orange: upregulated; blue: downregulated), normalized to the control group. (**B**–**D**) Volcano plots showing the distribution of significantly differentiated metabolites at 12 h, 48 h, and 96 h, respectively. Significantly differentiated metabolites were screened with FDR < 0.05 and |log_2_FC| > 1. *x*-axis: log-transformed fold change between groups; *y*-axis: −log10 *P* of group differences. Metabolites closer to the two ends of the *x*-axis have greater differential expression; different colors indicate up- or downregulated metabolites according to the threshold, and grey points indicate non-differentiated metabolites.

The bar chart of total number of differential metabolites results showed that, in the POS, the stressed groups exhibited 35, 12, and 45 significantly upregulated DAMs at 12 h, 48 h, and 96 h, respectively, alongside 56, 22, and 6 significantly downregulated DAMs at the corresponding time points (Figure 5A). In the NEG, 102, 4, and 173 DAMs were significantly upregulated at the three time points, respectively, whereas 79, 27 and 19 DAMs were significantly downregulated (Figure 6A).

Analysis of the volcano plot in positive ion mode (Figure 5B–D) revealed that the overall DAM expression profiles displayed a time-dependent downregulation-then-upregulation pattern at distinct time points post low-salinity acclimation and high ammonia nitrogen stress. Volcano plot analysis in negative ion mode (Figure 6B–D) revealed that at distinct time points post low-salinity acclimation and high ammonia nitrogen stress, the overall significantly DAMs exhibited an initial upregulation followed by downregulation, with a renewed upward trend at 96 h post-stress.

### 3.8. KEGG Enrichment Analysis of Significantly Differentiated Metabolites

To identify significant differential metabolites and analyze their metabolic pathways, the screening criteria were defined as VIP ≥ 1 (OPLS-DA) and *p* < 0.05 (*t*-test). Subsequently, the identified differential metabolites were mapped to reference pathways in the KEGG database.

After 12 h of exposure to high ammonia nitrogen stress under low-salinity acclimation conditions, the identified significantly differential metabolites were dominated by sphingosines and organic acids and their derivatives. Among these metabolites, norfloxacin and taurine both had VIP > 5, being the highly significant differential metabolites at this time point (Figures S5A and S6A). After 48 h exposure, the identified significantly differential metabolites were dominated by sphingophospholipids, polyphenols, and organic acids and their derivatives. Of these, 1-Palmitoyl-sn-glycero-3-phosphocholine, taurine, and pseudouridine all had VIP > 5, being the highly significant differential metabolites at this time point (Figures S5B and S6B). Low-salinity acclimation followed by high ammonia nitrogen stress for 96 h revealed that the significantly differential metabolites detected were primarily composed of organic acids and their derivatives, fatty acid esters of lipid molecules, and organic oxygen-containing compounds. Of these, taurine and betaine exhibited VIP > 5 and were identified as the most significant differential metabolites at this time point (Figures S5C and S6C).

**Figure 7:**
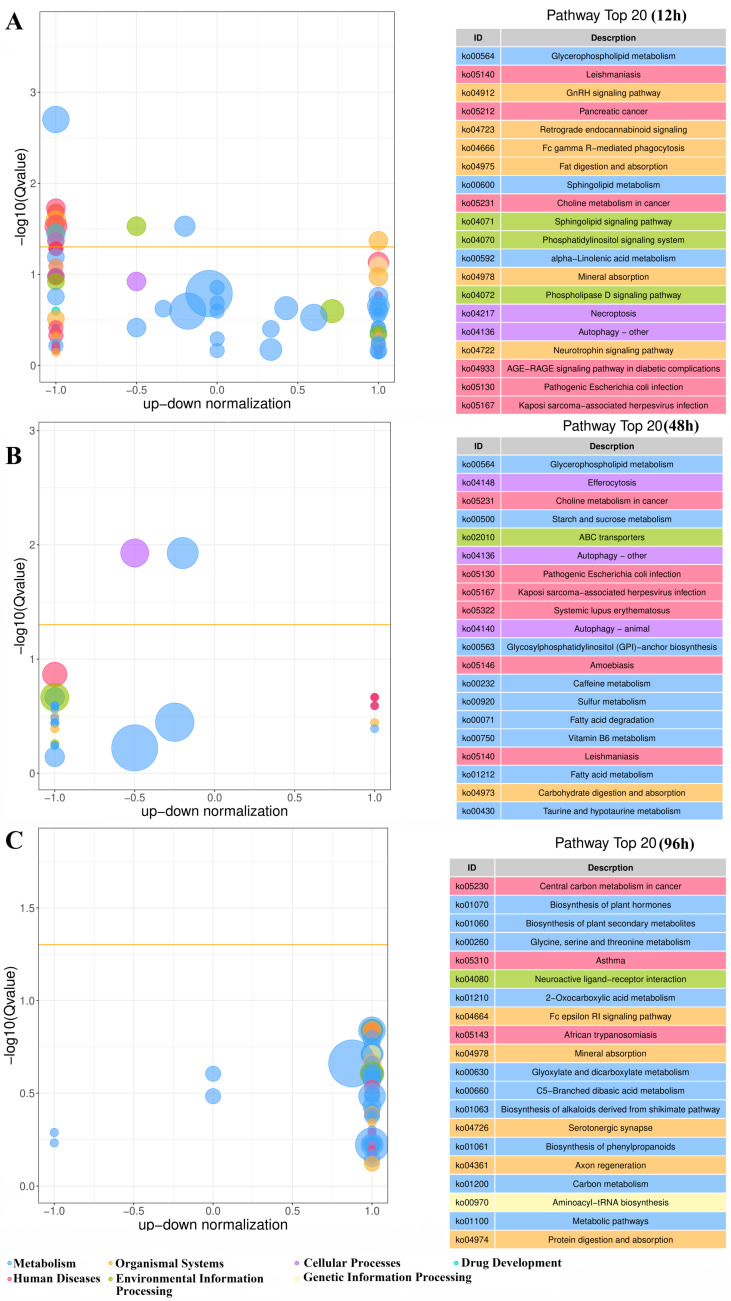
KEGG enrichment bubble plot. (**A**–**C**) show the KEGG pathway enrichment profiles after 12 h, 48 h, and 96 h of high ammonia nitrogen stress following low-salinity adaptation, respectively. Pathways were screened using a *p* < 0.05 threshold, selecting the top 20 pathways with the lowest Q-values. The *x*-axis represents the up-down normalization value (i.e., the ratio of the difference between the number of upregulated and downregulated differential metabolites to the total number of differential metabolites), indicating the overall trend of gene expression within the pathway. A positive *x*-axis value indicates a predominance of upregulated genes in the pathway, a negative value indicates a predominance of downregulated genes, and a value close to zero indicates a relatively balanced number of upregulated and downregulated genes. The *y*-axis displays −log10 (Q-value). Higher values correspond to smaller Q-values, indicating stronger enrichment. The yellow line represents the Q-value threshold of 0.05, signifying the statistical significance of pathway enrichment. Bubble size is generally proportional to the number of significantly differentially expressed genes in the pathway; larger bubbles indicate more genes undergoing significant regulation. Bubble color categorizes pathways. The right panel displays the top 20 pathways by Q-value.

In KEGG enrichment analysis, the integrated KEGG enrichment bubble plot of gill tissue demonstrated that under high ammonia nitrogen stress subsequent to low-salinity acclimation, the significantly perturbed metabolic pathways were predominantly associated with primary metabolic pathways, ABC transporter, cell membrane integrity-maintaining pathway, signaling pathway (e.g., glycerophospholipid metabolism), immune-related pathway (e.g., Leishmaniasis, Pathogenic E. coli Infection, Viral Stress Markers), and autophagic pathway (Figure 7).

### 3.9. Results of Integrated Analysis of Transcriptome and Metabolome

To better reveal the intrinsic regulatory relationship, the transcriptome and metabolome were combined to identify co-enriched KEGG pathways in which differentially expressed genes and differential metabolites were simultaneously enriched. In these common pathways, gene expression changes and metabolite accumulation changes can verify and complement each other, so as to more reliably reflect the real biological processes under ammonia nitrogen stress.

**Figure 8:**
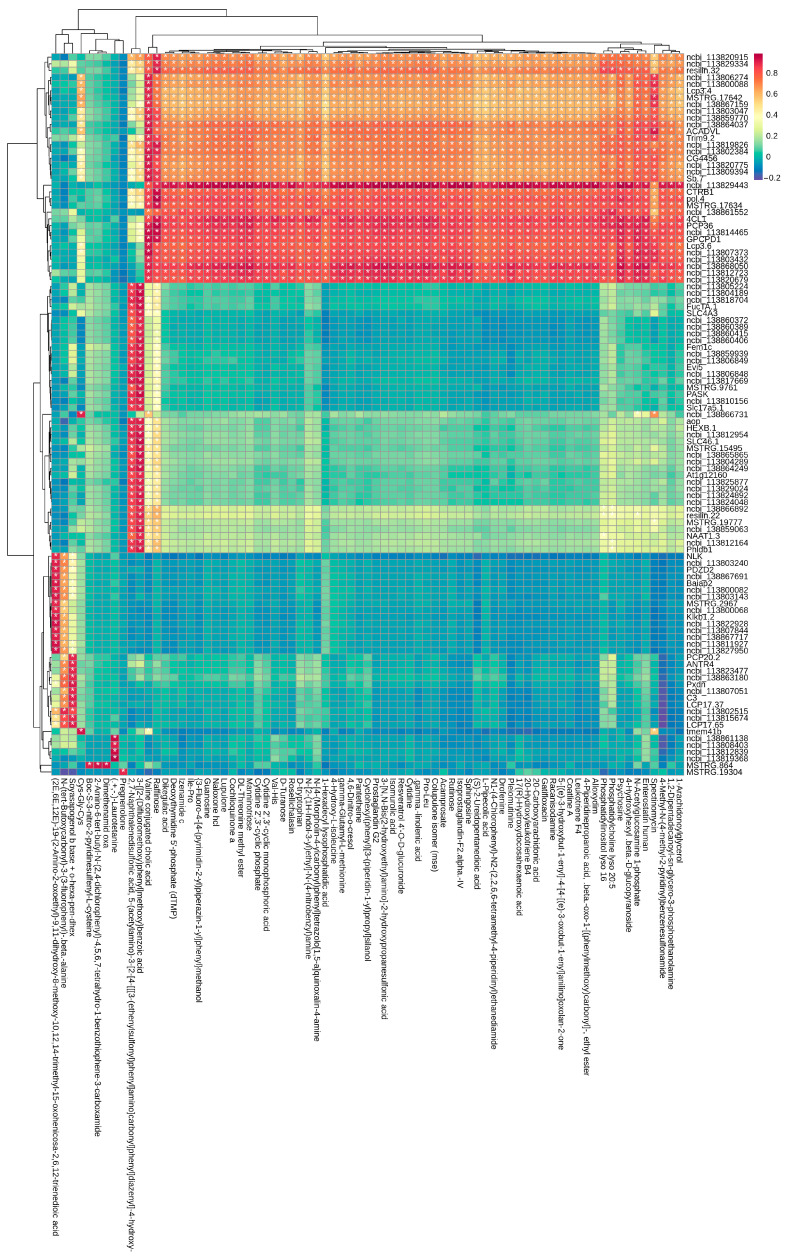
Heatmap of correlations between differentially expressed genes and metabolites. The top 250 differentially expressed genes and metabolites ranked by correlation coefficient are visualized as a heatmap; the *x*-axis displays metabolite names, while the *y*-axis shows NCBI IDs of differentially expressed genes. Gradient colors indicate the strength of correlation between genes and metabolites, with reddening colors signifying stronger correlations and bluer colors indicating weaker correlations. Correlation *p*-values are displayed using “*” symbols. A higher number of “*” indicates a more significant correlation between the gene and metabolite.

To further delineate the potential regulatory associations between differentially expressed genes (DEGs) and differentially accumulated metabolites (DAMs) in the gill tissues of *L. vannamei* in response to high ammonia nitrogen stress following low-salinity acclimation, we adopted a pathway-centric multi-omics integration strategy to correlate transcriptomic and metabolomic datasets. Gene–metabolite expression correlation coefficients were calculated across all experimental samples to construct a correlation heatmap (Figure 8).

The five core pathways were selected based on significant KEGG enrichment (*p* < 0.05), co-enrichment in transcriptome and metabolome, and inclusion of differentially expressed genes (|log_2_FC| ≥ 1, *p* < 0.05) and differentially accumulated metabolites (|log_2_FC| ≥ 1, *p* < 0.05, VIP ≥ 1) across multiple time points.

**Figure 9:**
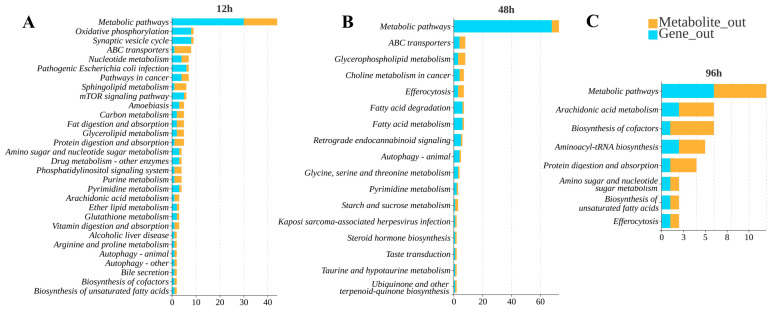
KEGG pathway enrichment bar chart. (**A**) Combined KEGG pathway diagram at the 12 h stress time point; (**B**) combined KEGG pathway diagram at the 48 h stress time point; (**C**) combined KEGG pathway analysis at the 96 h stress time point. This diagram is plotted based on the number of enriched genes/metabolites within each pathway. Each bar represents a pathway, with its length indicating the number of enriched genes/metabolites (blue denotes differentially expressed genes, orange denotes differentially expressed metabolites). The *x*-axis represents the number of differentially expressed genes or metabolites; the *y*-axis displays the pathway name. Legend in upper right corner: Gene_out denotes the number of genes enriched in this pathway within the analyzed gene set; Metabolite_out denotes the number of metabolites enriched in this pathway within the analyzed metabolite set.

Subsequently, these five core pathways were identified via combined transcriptomic and metabolomic analysis of high ammonia nitrogen stress in low-salt-adapted *L. vannamei*: autophagy pathway, metabolic pathway, fatty acid degradation and metabolism, ABC transporter, and immune-related pathway (Figure 9). Key genes and metabolites across distinct stress time points exhibited specific expression and accumulation profiles as follows.

At 12 h post-stress treatment, an integrated analysis of transcriptomic and metabolomic datasets identified a total of 37 co-enriched regulatory pathways, mainly including the metabolic pathway, the ABC transporter, autophagy and immune-related pathway. Among these, the metabolic pathway contained 30 key functional genes screened relative to the control group, primarily including UMP-CMP kinase-like isoform X1 (*CMPK*), adenosine deaminase 2 (*ada2*)*,* and uridine-cytidine kinase-like 1 isoform X1 (*UCKL1*) (which regulate nucleotide metabolism), chitotriosidase-1-like (*Cht10*) (which mediates amino sugar and nucleotide sugar metabolism), and *ACADM* (which modulates the degradation of valine, leucine and isoleucine). In the ABC transporter, one key functional gene (*ABCD3*) was identified, while the autophagy also contained one key functional gene (*atg101*). The key genes in the immune-related pathways mainly included *Act5C*, *VhaSFD* and V-type proton ATPase subunit C-like (*atp6v1c1a*). A total of 80 significantly differential metabolites that showed strong correlations with differentially expressed genes were screened, mainly including norfloxacin, taurine, guanosine, leucine, and other metabolites.

At 48 h post-stress treatment, a total of 17 co-enriched regulatory pathways were identified, including the ABC transporter, global metabolic pathway, glycerophospholipid metabolism, fatty acid metabolism and animal autophagy. Among these, the metabolic pathway contained 68 key functional genes, represented by carbohydrate sulfotransferase 12 (*Chst12*), glutamate-cysteine ligase catalytic subunit-like (*GCLC*), glutamine synthetase (*Gs2*), and trifunctional enzyme subunit alpha (*HADHA*). Four key genes were identified in the ABC transporter, namely multidrug resistance-associated protein 1-like isoform X2 (*Abcc1*), *ABCB10*, *Abcd3*, and ATP-binding cassette sub-family G member 5-like(*ABCG5*); six key genes were found in the fatty acid metabolism, including *ACAT2*, *HADHA*, very long-chain specific acyl-CoA dehydrogenase (*ACADVL*), carnitine O-palmitoyltransferase 1 (*Cpt1a*), *Acads*, and *ACADM*; and four key genes were detected in the autophagy, specifically ras-related GTP-binding protein C-like (*Rragc*), transcription factor SPT20 homolog isoform X1 (*SUPT20H*), microtubule-associated proteins 1A/1B light chain 3C-like isoform X1 (*MAP1LC3C*) and pleckstrin homology domain-containing family M member 1-like (*plekhm3*). A total of 34 significantly differential metabolites that showed strong correlations with differentially expressed genes were screened, represented by 1-palmitoyl-sn-glycero-3-phosphocholine, taurine, and pseudouridine.

At 96 h post-stress treatment, only eight common pathways were identified, including the global metabolic pathway, arachidonic acid metabolism, protein digestion and absorption, amino sugar and nucleotide sugar metabolism, and biosynthesis of unsaturated fatty acids. Among these, the basic metabolic pathway involved six key genes, namely *Mocs2B*, *Cyp2b1*, Dolichyl-diphosphooligosaccharide-protein glycosyltransferase subunit 2 (*RPN2*), stearoyl-CoA desaturase 5-like (*SCD5*), *ACADM*, and *Cht10*. Six significantly differential metabolites that showed strong correlations with differentially expressed genes were screened, including taurine and betaine; a total of 24 significantly differential metabolites that showed strong correlations with differentially expressed genes were identified across all the pathways.

To further clarify the regulatory relationships between key DEGs and DAMs and their roles in stress-response networks, we identified gene–metabolite interactions in core co-enriched pathways at different time points post high ammonia nitrogen stress (Table 5). At 12 h, *ABCD3* in the ABC transporter pathway was correlated with norfloxacin, guanosine, taurine, and leucine, while *CMPK*, *ada2*, and *UCKL1* in the metabolic pathway were associated with guanosine, and *ACADM* was linked to leucine. At 48 h, ABC transporter genes (*Abcc1*, *Abcd3*, *ABCG5*, *ABCB10*) maintained correlations with taurine; fatty acid metabolism genes (*Acads*, *ACAT2*, *ACADM*, *ACADVL*, *Cpt1a*) were significantly correlated with 1-palmitoyl-sn-glycero-3-phosphocholine; and metabolic pathway genes (*Chst12*, *GCLC*, *Gs2*, *HADHA*) were associated with taurine and pseudouridine. At 96 h, *ACADM*, *SCD5*, and other genes in the metabolic pathway were closely associated with taurine and betaine. These interaction pairs form a coordinated regulatory network.

## 4. Discussion

The integrated transcriptomic and metabolomic analyses revealed that from an overall dynamic trend, the number of co-enriched pathways and significantly differentially expressed genes both reached the maximum level at 48 h. These findings imply that shrimp initiated comprehensive transcriptomic and metabolic reprogramming to defend against high ammonia nitrogen stress following low-salinity acclimation. Accordingly, 48 h may represent a critical window for the activation of stress-responsive regulatory pathways. From the perspective of genetic breeding, it can be speculated that the dynamic molecular alterations observed at 48 h and 96 h contribute to the identification of key genes and metabolites associated with ammonia nitrogen tolerance under low-salinity conditions. These molecules may serve as potential candidate biomarkers for stress tolerance. Upon further experimental verification, such biomarkers could be utilized to evaluate stress tolerance capacity, which may provide a theoretical foundation for the future selective breeding of shrimp varieties with enhanced resistance to low-salinity and high ammonia nitrogen environments. To further clarify the core biological processes underlying these stress responses and molecular changes, five key pathways were jointly identified by the transcriptomic and metabolomic analysis. These pathways comprise ABC transporter, autophagy, immune-related pathway, basic metabolic pathway, and fatty acid degradation and metabolism. Detailed interpretations of the key differentially expressed genes and corresponding pathways are provided below.

Firstly, the metabolic pathway, as one of the most prominent pathways in this study, encompasses key genes including *CMPK* and *ada2*, which may be involved in nucleotide metabolism, and *ACADM*, which is putatively associated with the degradation of valine, leucine, and isoleucine. Among these, ammonia nitrogen stress disrupts amino acid metabolism, nucleotide metabolism, and lipid metabolism, with significant upregulation of *CMPK* and *ada2* gene expression [30]; activation of leucine metabolism may contribute to protein synthesis and cell proliferation, alleviating the inhibitory effect of ammonia nitrogen stress on tissue repair [31]. Moreover, under ammonia nitrogen stress, nucleotide metabolism provides the material basis for cell proliferation and differentiation [32]. This pathway synergizes with leucine metabolism to maintain the equilibrium of protein and nucleic acid synthesis, thereby helping sustain the organism’s capacity for growth and repair under stressful conditions [33]. Although the aforementioned studies share similarities with the present research, they did not investigate or describe multiple stress time points. In transcriptomic studies of *Penaeus monodon* and *Scylla paramamosain* under ammonia stress, amino acid, nucleotide, and lipid metabolism were also commonly disturbed, which is consistent with our results. Nevertheless, those studies did not consider the effect of prior low-salinity acclimation; thus, our study provides a more realistic simulation for actual low-salinity farming systems [34,35]. In summary, metabolic pathways and their key genes may serve crucial roles in mitigating ammonia nitrogen oxidation stress.

Secondly, the enrichment of the autophagy suggests that organisms may achieve that organisms achieve self-repair of stress-induced damage by degrading damaged cellular components [36]. Previous studies have indicated that ammonia nitrogen stress significantly induces the expression of autophagy-related genes (e.g., autophagy related 3 (*ATG3*), autophagy related 4 (*ATG4*), autophagy related 10 (*ATG10*)) in *L*. *vannamei* [37]. Concurrently, ammonia nitrogen stress may activate autophagy by regulating the miRNA-autophagy pathway axis (e.g., *pva-miR-252* modulating the phosphatidylinositol 3-kinase (*PvPI3K*) gene), thereby potentially enhancing antioxidant capacity to counteract ammonia nitrogen-induced oxidative stress damage [38]. This study similarly identified the autophagy under high ammonia nitrogen stress after low-salinity acclimation, which is consistent with previous findings. It is worth noting that our screening results identified only *atg101* as a key differentially expressed gene involved in this pathway. As a widespread environmental stressor in aquaculture systems, ammonia nitrogen triggers cellular stress responses, which can activate AMPK and suppress the mTOR signaling pathway. This in turn promotes the assembly and activation of the ULK1/2-ATG13-FIP200 complex, a core module containing multiple ATG family proteins that initiates autophagy [39,40]. Members of the ATG gene family participate in the core autophagy machinery to sustain cellular homeostasis and development, modulate cellular metabolism and survival, and regulate diverse biological processes including embryonic development, organogenesis (e.g., early zebrafish development and crustacean hematopoiesis), and immune defense [39]. To the best of our knowledge, *atg101* has not previously been recognized as a key differentially expressed gene involved in the autophagy pathway under high ammonia nitrogen stress. Validation of this key gene may provide novel molecular targets and theoretical support for breeding aquatic animals with enhanced ammonia nitrogen tolerance, thereby bridging the current knowledge gap regarding autophagy-related molecular markers for improving ammonia nitrogen resistance in aquaculture species.

Thirdly, the ABC transporter pathway was particularly prominent in the 48 h and 96 h treatment groups. Previous studies have indicated that ammonia nitrogen stress may induce toxic substance accumulation and ionic imbalance in *L. vannamei* [41]. The ABC transporter may enhance the transmembrane transport and excretion of ammonia nitrogen metabolic intermediates and toxic substances by activating its family members (such as genes from the *ABCC* and *ABCF* subfamilies) [42]. Concurrently, this pathway mitigates the disruption of the intracellular environment induced by ammonia nitrogen stress by regulating ion channel homeostasis [43]. In this study, the ABC transporter exhibited significant enrichment, and the significantly differentially expressed genes identified also belonged to ABC transporter subfamilies (e.g., *ABCD3*, *ABCB10*). This supports the view that ABC transporters may participate in regulating ion channel homeostasis and facilitate the efflux of toxic substances in gill tissues.

Fourth, the fatty acid degradation and metabolism constitute one of the key energy metabolism pathways, and the key differentially expressed genes identified in this pathway are *ACAT2*, *Cpt1a*, and *Acads*. Research indicates that under combined hypoosmotic-ammonia nitrogen stress, the choline metabolic pathway may act synergistically with energy metabolism pathways such as fatty acid metabolism and the tricarboxylic acid cycle [41]. This provides the energy required for the normal expression of osmoregulatory genes (e.g., *NKA*, RCC1, and BTB domain-containing protein claret (*CA*)) in gill tissues and for ion transport processes, thereby maintaining intracellular and extracellular osmotic equilibrium [44]. Similarly, under high ammonia nitrogen stress, energy metabolism pathways in *L*. *vannamei* are also inhibited [40,45]. Among these, *Acad* and *ACTAT* have been reported in previous low-salinity stress studies to participate in regulating lipid synthesis and degradation to maintain energy balance [46,47]. This indicates that whether under low-salinity or high ammonia nitrogen stress, combined low-salinity and ammonia nitrogen stress, or ammonia nitrogen stress following low-salinity acclimation as in this study, all three conditions impact fatty acid degradation and metabolic pathway. In ammonia-stress transcriptomes of *L. vannamei* and *Portunus trituberculatus*, fatty acid degradation and beta-oxidation were commonly reprogrammed to meet extra energy demand. Our study further confirmed this pattern under a more complex condition (low-salinity acclimation followed by ammonia exposure), suggesting a universal energy compensatory mechanism in crustaceans [48,49].

Fifth, the immune-related pathways associated with immune defense in this study include the pathogenic *Escherichia coli* infection pathway and the viral stress response pathway, involving key genes such as *Act5C* and *VhaSFD*. The enrichment of these pathways and genes indicates that organisms activate broad-spectrum immune defense mechanisms in the early stages of stress to counteract immune stress induced by ammonia nitrogen toxicity. Previous studies have indicated that ammonia nitrogen stress significantly disrupts innate immune signaling in *L*. *vannamei*, exacerbating the risk of pathogen infection by suppressing the activation of antimicrobial-associated pathways or inducing inflammatory responses [44]. Concurrently, ammonia nitrogen stress increases shrimp susceptibility to viruses, thereby activating viral stress response pathways to generate a comprehensive immune response with both antibacterial and antiviral functions [50]. Simultaneously, *Act5C*, as an actin gene, is involved in cytoskeletal remodeling and immune cell migration, enhancing shrimp resistance to pathogens and environmental stressors [51].

The results of the joint analysis indicate that the significantly differential metabolites identified in this study mainly include taurine, guanosine, 1-palmitoyl-sn-glycero-3-phosphocholine, pseudouridine, and betaine. These metabolites play key roles in maintaining gill function, osmotic balance, and ammonia detoxification. As the main respiratory and osmoregulatory organ, the gill relies on betaine and taurine to stabilize osmotic pressure and cell structure under low-salinity stress, while the phospholipid helps maintain gill cell membrane fluidity to support normal ion transport and ammonia excretion. In crustaceans, several studies have reported that betaine, as an important organic osmolyte, can maintain cell volume and stabilize protein structure under combined low-salinity and ammonia nitrogen stress [52]. Taurine can alleviate oxidative damage and enhance stress resistance in shrimp by participating in antioxidant processes, osmotic balance, and ammonia metabolism detoxification, and such functions have been supported by relevant research [53]. As a key glycerophospholipid in cell membranes, 1-palmitoyl-sn-glycero-3-phosphocholine contributes to maintaining membrane integrity and fluidity under environmental stress, but related knowledge is mostly concentrated in vertebrates such as teleost fish [54]. Notably, the integrated physiological functions and regulatory mechanisms of these metabolites in response to high ammonia nitrogen stress after low-salinity acclimation remain rarely systematically elucidated or directly verified in crustaceans. The present study for the first time identified the significant changes of these key metabolites in low-salinity-acclimated *L. vannamei*. In concrete terms, betaine acts as a critical osmoprotectant during low-salinity acclimation, and its synthetic pathway is significantly upregulated under such conditions [55]. The present study also focused on low-salinity acclimation, and betaine showed significant upregulation after 96 h of stress. This suggests that subsequent ammonia nitrogen stress following low-salinity acclimation further promotes betaine accumulation, which represents a partial extension of previous findings. Among these, taurine emerged as the most critical significantly differential metabolite, showing marked enrichment at 12 h, 48 h, and 96 h post-stress. Previous studies have indicated that taurine supplementation mitigates excessive NKA activation in shrimp larvae under low-salinity stress, thereby enhancing their osmoregulatory capacity [56]. Under ammonia nitrogen stress, taurine participates in antioxidant and detoxification metabolism, thereby reducing oxidative stress-induced damage [57]. In this study, taurine was involved in metabolic pathways and ABC transporter pathways, showing an enrichment trend of initial upregulation, subsequent downregulation, and then re-upregulation across the three stress time points. This phenomenon may be attributed to the following: at 12 h post-stress, shrimp initiate rapid emergency responses; as a critical antioxidant and cell membrane protector, taurine is synthesized in large quantities and participates in ammonia detoxification; at 48 h post-stress, the precursors or energy required for taurine synthesis are consumed by more central ammonia detoxification pathways; at 96 h post-stress, the organisms re-accumulate taurine to adapt to prolonged stress.

It should be emphasized that taurine responds to multiple environmental stressors rather than being uniquely specific to ammonia nitrogen stress. Therefore, we speculate that taurine, especially when combined with other reliable biomarkers, holds promise as a candidate indicator for evaluating stress levels in white shrimp under high ammonia nitrogen stress following low-salinity acclimation. Further studies, including validation with larger sample sizes, gradient ammonia nitrogen exposure, multiple stress comparisons, and feeding trials of taurine supplementation, are needed to verify its specificity, stability, and practical application value as a stress biomarker.

Overall, the integrated analysis reveals the coordinated regulatory mechanisms underlying shrimp responses to ammonia stress. Ammonia toxicity initially induces metabolic disturbance and energy deficiency in gill tissue, driving a rapid reallocation of energy toward stress defense. These pathways then form an integrated and coordinated regulatory network: fatty acid degradation and global metabolic pathways provide sufficient ATP and metabolic substrates to support the overall defense process. The resulting metabolic stress and cellular damage further trigger autophagy, which clears damaged organelles and maintains intracellular homeostasis while alleviating ammonia-induced cell injury. The ABC transporter pathway promotes the efflux of toxic metabolites and regulates ion homeostasis to reduce intracellular ammonia toxicity, and immune-related pathways are simultaneously activated to counteract immunosuppression and secondary pathogen invasion. These pathways interact systematically rather than function independently: energy metabolism fuels autophagy, ABC transporter activity, and immune responses, while cellular repair, toxin excretion, and immune defense in turn stabilize the internal environment for sustained metabolic homeostasis. This synergistic mechanism allows shrimp to effectively cope with high ammonia nitrogen stress under low-salinity conditions.

This study systematically reveals the molecular responses of shrimp to high ammonia nitrogen stress after low-salinity acclimation, and identifies several key genes and metabolites closely related to stress tolerance. These molecular indicators can be used to evaluate the physiological status and stress levels of shrimp under low-salinity culture conditions. In addition, these key molecules can be applied as candidate biomarkers for the screening and breeding of ammonia-tolerant and low-salinity-adapted shrimp strains, which will help to breed excellent varieties with strong environmental adaptability and high survival rate. Meanwhile, the results of this study can provide a theoretical reference for the rational control of ammonia nitrogen concentration in low-salinity culture systems, scientific feeding management, and the reduction of stress-related risks in practical shrimp culture.

Notably, several potential limitations should be acknowledged. First, only gill tissues were analyzed; other tissues may exhibit distinct responses to stress. Second, although randomization and standardization were applied, some unavoidable variation in individual shrimp may introduce minor bias. Third, most functional annotations of the identified genes and proteins were predicted based on sequence homology, and their functions were mainly inferred from model organisms including humans. Thus, caution is needed in functional interpretation in marine invertebrates such as *L. vannamei.* Finally, the molecular mechanisms revealed by transcriptomic and metabolomic analyses require further verification using molecular biology techniques such as gene knockdown. Additionally, the present study used the 96 h LC_50_ to simulate acute ammonia spikes in aquaculture. Under such severe acute stress, the observed responses may reflect physiological disruption rather than mild adaptation. Results under sublethal or chronic ammonia exposure would likely differ, representing more gradual adaptive changes. Furthermore, qRT-PCR was performed using pooled samples, which may reduce biological variation and hinder direct comparison with individual-based RNA-seq data. Three technical replicates were included to ensure reliability. These limitations do not affect the main conclusions but provide directions for future research.

## 5. Conclusions

This study employed an integrated transcriptomics and metabolomics approach to investigate the molecular mechanisms underlying the response of *L. vannamei* to high ammonia nitrogen stress (42.32 mg/L) following low-salinity acclimation (5‰), with the aim of identifying key genes, metabolites, and pathways. Transcriptomic analysis identified 352, 802, and 140 differentially expressed genes (DEGs) at the three time points, respectively. Metabolomic analysis detected significant changes in differential metabolites (DAMs) across both ion modes, primarily involving lipids, amino acid derivatives, and other related compounds. Multi-omics integration analysis identified five core regulatory pathways: ABC transporter, autophagy, fatty acid degradation and metabolism, immune-related pathway, and metabolic pathway. Key genes in these pathways and their corresponding metabolites were further characterized. This study deepens the understanding of molecular mechanisms underlying the response of *L. vannamei* to high ammonia nitrogen stress under low-salinity conditions, and provides a theoretical basis for sustainable aquaculture in estuarine areas. These results can guide aquaculture practitioners to optimize feeding management, water quality regulation, and ammonia nitrogen control, thus reducing stress-induced mortality and enhancing aquaculture efficiency.

## Figures and Tables

**Table 1:** Primers used for qRT-PCR.

Primer Name	Sequence (5′ to 3′)	Length (bp)
*Acads*-F	CAAGCAGAGCATGTACCCCA	20
*Acads*-R	GTAGCACAACCCCTGCTGAT	20
*ACAT2*-F	GGGACTGGGACTGTAACTGC	20
*ACAT2*-R	CCATAACGGCTGGGTCAACT	20
*atg101*-F	GCAGTTGGGACCATAGGCTT	20
*atg101*-R	TTCCAGAGCAACCTGTCCAC	20
*atp6v1c1a*-F	TCGTTGGAGCTGCTATGGGTA	21
*atp6v1c1a*-R	CCTCGCAGAAGATGACCGAG	20
*HSP90AA1*-F	CCACGACACTGGCGAACC	18
*HSP90AA1*-R	GCCTACATCTTCGATTTTGGGCT	23
*Mocs2B*-F	ACATGGAAGCAGCACTGACA	20
*Mocs2B*-R	GTCGTTCCTCGATGCCTTCT	20
*Cyp2b1*-F	GGGCACCGTCCTTAGTTTCA	20
*Cyp2b1*-R	CTGGCCGACCTTTTTCTCCA	20
*GPx*-F	CAACCAATTCGGGAAGCAAGAG	22
*GPx*-R	GGTGTTCATTCTCGCCGTTC	20
*GstD1*-F	CAGCACAGCGTTCCTACCA	19
*GstD1*-R	AACATCACGGGATACGCATACT	22
*GCLC*-F	ACGAATTTCTTCAGGCTGGGT	21
*GCLC*-R	TCTTCAACGAAGGGACGTGG	20
*β-actin*-F	GCCCATCTACGAGGGATA	18
*β-actin*-R	GGTGGTCGTGAAGGTGTAG	19

**Table 2:** RNA-seq sequencing data.

Sample	RawData (bp)	CleanData (%)	LowQuality (%)	GC (%)	Q20 (%)	Q30 (%)	Total_Mapped (%)
12 h-CK	7,343,678,750	99.94	0.00	41.62	99.41	97.68	88.70
48 h-CK	6,673,405,850	99.93	0.00	43.15	99.42	97.68	89.08
96 h-CK	7,321,914,500	99.94	0.00	43.35	99.31	97.48	88.49
12 h	7,094,962,750	99.92	0.00	44.37	99.36	97.58	87.87
48 h	6,992,928,000	99.94	0.00	41.66	99.45	97.90	86.24
96 h	6,731,374,350	99.92	0.00	41.78	99.36	97.69	87.96

**Table 3:** Representative genes with significant differential expression.

Accession Number Gene Name Description			log2ratio		
			12 h	48 h	96 h
Fatty acid degradation and metabolism					
ncbi_113829792	*Acads*	short-chain specific acyl-CoA dehydrogenase, mitochondrial-like	0.10	1.32	−0.62
ncbi_113809375	*ACAT2*	acetyl-CoA acetyltransferase, cytosolic-like	0.38	−3.69	−1.43
ncbi_113805029	*HADHA*	trifunctional enzyme subunit alpha, mitochondrial-like	0.31	1.85	−0.64
ncbi_113821199	*Cpt1a*	carnitine O-palmitoyltransferase 1, liver isoform-like	0.46	−2.68	0.10
ncbi_113813193	*ACADVL*	very long-chain specific acyl-CoA dehydrogenase, mitochondrial-like	0.36	−2.86	−0.18
ncbi_113816547	*Baldspot*	very long chain fatty acid elongase 6-like isoform X1	2.47	1.24	−0.43
Autophagy ncbi_113821166	*VPS41*	vacuolar protein sorting-associated protein 41 homolog isoform X1	0.26	0.41	−1.40
ncbi_113816298	*atg101*	autophagy-related protein 101-like	1.35	−0.15	−0.17
ncbi_138859707	*MAP1LC3C*	microtubule-associated proteins 1A/1B light chain 3C-like isoform X1	−0.35	−2.09	0.71
ncbi_113806572	*Rragc*	ras-related GTP-binding protein C-like	0.46	1.27	−0.30
ncbi_113814862	*SUPT20H*	transcription factor SPT20 homolog isoform X1	−0.40	1.70	−0.87
ncbi_138860752	*plekhm3*	pleckstrin homology domain-containing family M member 1-like	−0.24	1.16	−0.30
Immune-related genes					
ncbi_113803489	*VhaSFD*	V-type proton ATPase subunit H-like	1.69	0.02	−0.39
ncbi_113822054	*ATP6V1B*	V-type proton ATPase subunit B-like, partial	1.99	−0.67	−1.27
ncbi_113828445	*atp6v1c1a*	V-type proton ATPase subunit C-like	2.00	−0.33	−0.59
ncbi_113809213	*Vha36-* *1*	V-type proton ATPase subunit D-like	1.92	−0.17	−0.16
ncbi_113821104	*VHA16*	V-type proton ATPase 16 kDa proteolipid subunit	2.42	−0.33	−0.52
ncbi_113826027	*Atp6v0b*	V-type proton ATPase 21 kDa proteolipid subunit-like	2.24	−0.04	−0.25
ncbi_113813026	*Act5C*	actin-5C-like	2.22	0.44	0.45
ncbi_113824160	*THBS4*	cartilage oligomeric matrix protein-like	−11.2	0.59	4.09
ncbi_113814443	*LanB2*	laminin subunit gamma-1-like	1.34	−1.07	0.42
ncbi_113824242	*ab*	zinc finger and BTB domain-containing protein 7A-like	−0.34	0.87	−1.26
ncbi_113805260	*hsp70*	heat shock 70 kDa protein cognate 4-like	−2.93	2.50	−4.57
ncbi_113802271	*hsp70*	heat shock protein	−1.75	2.10	−1.91
ncbi_113825748	*HSPA1A*	heat shock 70 kDa protein cognate 4-like	−1.64	2.90	−5.37
ncbi_113806042	*HSP90AA1*	LOW QUALITY PROTEIN: heat shock protein HSP 90-alpha-like	−0.24	2.21	−1.91
ncbi_113806043	*HSP90AA1*	heat shock protein HSP 90-alpha isoform X4	−0.89	2.50	−1.07
PPAR signaling pathway		NADP-dependent malic enzyme-like isoform X1	2.03	1.82	0.46
ncbi_113811598	*ME1*				
ncbi_138866703	*ACADM*	medium-chain specific acyl-CoA dehydrogenase, mitochondrial-like	−14.34	14.36	14.28
ncbi_113822166	*SCD5*	stearoyl-CoA desaturase 5-like chitotriosidase-1-like	−0.36 0.28	−1.10 −2.57	−1.67 4.99
Metabolic pathways					
MSTRG.12598	*Cht10*				
ncbi_113802125	*Mocs2B*	molybdopterin synthase catalytic subunit-like	−0.49	0.23	−1.33
ncbi_113812867	*Cyp2b1*	cytochrome P450 2U1-like	1.93	−0.53	−2.12
ncbi_113817801	*RPN2*	Dolichyl-diphosphooligosaccharide–protein glycosyltransferase subunit 2	0.24	−0.006	−1.05
ncbi_113805442	*UCKL1*	uridine-cytidine kinase-like 1 isoform X1	2.16	0.95	−0.66
ncbi_113816434	*CMPK*	UMP-CMP kinase-like isoform X1	2.57	2.85	−0.12
ncbi_113821421	*DLD*	dihydrolipoyl dehydrogenase, mitochondrial-like	0.10	1.14	−0.17
ncbi_113816684	*GPx*	GPX3	1.85	−1.89	0.04
ncbi_113817380	*Dlat*	dihydrolipoyllysine-residue acetyltransferase component of pyruvate dehydrogenase complex, mitochondrial-like	0.24	1.69	−0.14
ncbi_113821256	*GstD1*	LOW QUALITY PROTEIN: glutathione S-transferase 1-like	−1.02	3.33	1.39
ncbi_113816845	*ca2*	carbonic anhydrase 1-like	1.15	2.44	−0.71
ncbi_113806294	*Glo1*	Lactoylglutathione lyase	−0.12	3.80	0.73
ncbi_113803894	*Gs2*	glutamine synthetase	0.72	−2.47	0.56
ncbi_113829896	*CAT*	catalase-like isoform X1	0.43	−1.84	0.22
ncbi_113802161	*GCLC*	glutamate–cysteine ligase catalytic subunit-like	−0.29	−2.86	0.7
ncbi_113813424	*HEXB*	beta-hexosaminidase subunit beta-like	−0.20	−2.76	0.41
ncbi_138867462	*Hexa*	beta-hexosaminidase subunit beta-like	−0.29	−4.38	−0.13
ABC transporters ncbi_138864386	*ABCD3*	ATP-binding cassette sub-family D member 3-like	2.60	0.96	−1.26
ncbi_113807842	*ABCB10*	ATP-binding cassette sub-family B member 10, mitochondrial-like isoform X1	0.29	1.28	−0.18
ncbi_113820857	*Abcd3*	ATP-binding cassette sub-family D member 3-like	1.49	1.37	−0.21
ncbi_113805021	*Abcc1*	multidrug resistance-associated protein 1-like isoform X2	−0.68	4.21	0.50
ncbi_113828957	*ABCG5*	ATP-binding cassette sub-family G member 5-like	−0.71	−1.16	−0.31

**Table 4:** Metabolite identification results.

Type	All	Known Metabolite	Unknown Metabolite
POS	10,369	3229	7140
NEG	11,762	3599	8163

**Table 5:** Integrated analysis of key DEGs and key DAM_S_.

Time	Pathway	Key DEGs	Key DAM_S_
12 h	Nucleotide metabolism	*CMPK*, *ada2*, *UCKL1*	guanosine
	Valine, leucine, and isoleucine degradation	*ACADM*	leucine
	ABC transporter pathway	*ABCD3*	norfloxacin, guanosine, taurine, leucine
48 h	metabolic pathways	*Chst12*, *GCLC*, *Gs2*, *HADHA*	taurine, pseudouridine,
	ABC transporter pathway	*Abcc1*, *Abcd3*, *ABCG5*, *ABCB10*	taurine
	Fatty acid metabolism pathway	*Acads*, *ACAT2*, *ACADM*, *ACADVL*, *Cpt1a*	1-palmitoyl-sn-glycero-3-phosphocholine
96 h	metabolic pathways	*Mocs2B*, *Cyp2b1*, *RPN2*, *SCD5*, *ACADM*, *Cht10*	taurine, betaine

## Data Availability

The datasets generated and analyzed during the current study are available from the corresponding author upon reasonable request. Transcriptomic data have been deposited in the NCBI Sequence Read Archive (SRA) under BioProject accession [PRJNA1434427]. The data are publicly available at: https://www.ncbi.nlm.nih.gov/bioproject/PRJNA1434427 (accessed on 2 April 2026). Metabolomic data have been deposited in the MetaboLights database under study accession [MTBLS14054]. The data are publicly available at: https://www.ebi.ac.uk/metabolights/MTBLS14054 (accessed on 2 April 2026).

## Supplementary Materials

The following supporting information can be downloaded at: https://www.mdpi.com/article/10.3390/biology15080612/s1, Figure S1: Transcriptome analysis quality assessment; Figure S2: Gene expression abundance distribution profile; Figure S3: qRT-PCR validation of representative differentially expressed genes (DEGs); Figure S4: Metabolome correlation heatmap; Figure S5: VIP score plots of differentially expressed metabolites in positive ion; Figure S6: Statistical plot of VIP values for differentially expressed metabolites in negative ion mode.
