# Supplementary material for: Integrated Analysis of Transcriptome and Metabolome Reveals Molecular Responses to Ammonia Stress in the Gills of Litopenaeus vannamei Under Low-Salinity Conditions
Source: Biology (Basel). 2026 Apr 13;15(8):612. doi: 10.3390/biology15080612 (PMC13113159; doi:10.3390/biology15080612)
Supplement: Supplementary file 1 [file biology-15-00612-s001.zip › Supplementary File.pdf]

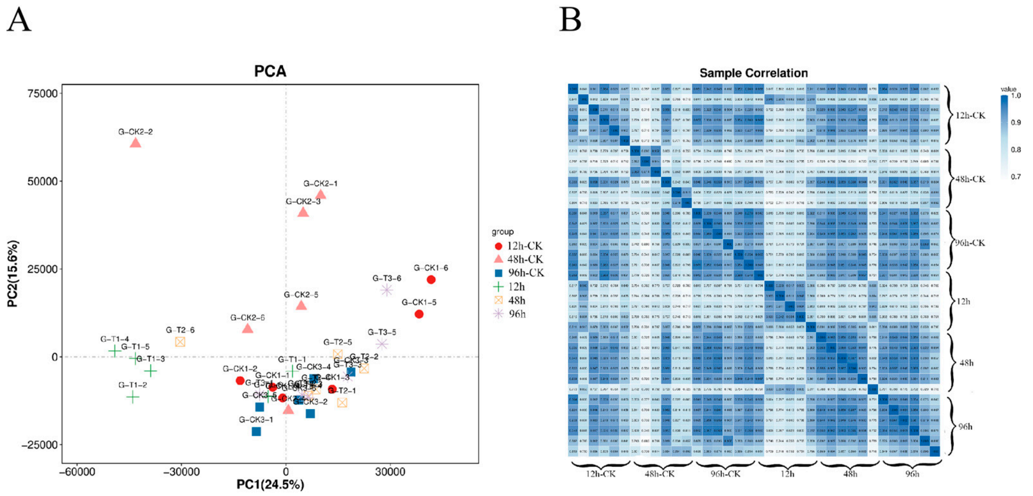

Figure S1. Transcriptome Analysis Quality Assessment. (A) Transcriptome PCA analysis. The Y-axis labels 12h, 48h, 96h represent the experimental group subjected to high ammonia nitrogen stress under low-salinity at three time points. The labels 12h-CK, 48h-CK, 96h-CK represent the control groups held in 5‰ salinity aquaculture water without high ammonia nitrogen stress at the corresponding time points. (B) Transcriptome Correlation Heatmap. The Y-axis labels 12h, 48h, and 96h represent the experimental groups subjected to high ammonia nitrogen stress under low-salinity at corresponding time points. The labels 12h-CK, 48h-CK, and 96h-CK represent the control groups held in 5‰ salinity aquaculture water without high ammonia nitrogen stress at the corresponding time points. The expression levels of each pair of samples were extracted to calculate the Pearson correlation coefficient between every two samples. These coefficients were then visually presented in the form of a heatmap to reflect the correlation between any two samples, which allows for the evaluation of the repeatability of intra-group replicate samples.

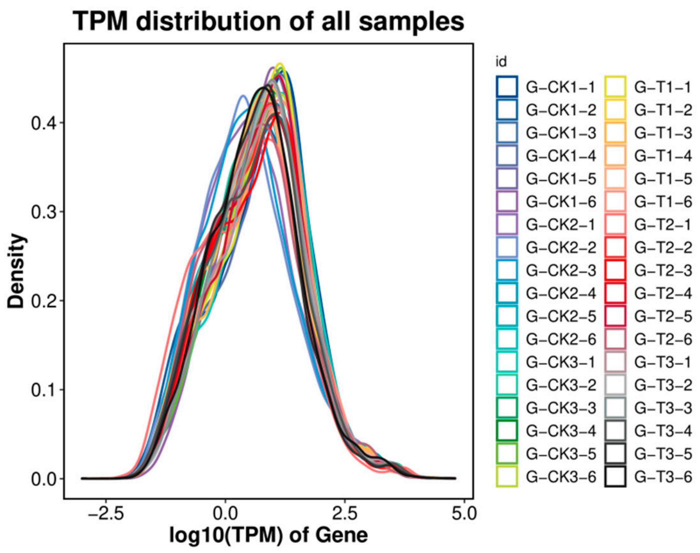

Figure S2. Gene Expression Abundance Distribution Profile. The X-axis represents log10(TPM), where higher values indicate higher gene expression levels. The Y-axis denotes gene

abundance, calculated as the proportion of genes with the corresponding expression level relative to the total number of detected genes. Each colored curve corresponds to an individual sample. In the experimental design, both the control and treatment groups included six biological replicates at each time point. G-CK represents the control group, with CK1, CK2, and CK3 corresponding to the 12h, 48h, and 96h control time points, respectively; G-T represents the treatment group, with T1, T2, and T3 corresponding to the 12h, 48h, and 96 h treatment time points, respectively. The peak of each distribution curve indicates the dominant expression range where genes are most concentrated across all samples, reflecting the typical transcriptional abundance level of the dataset.

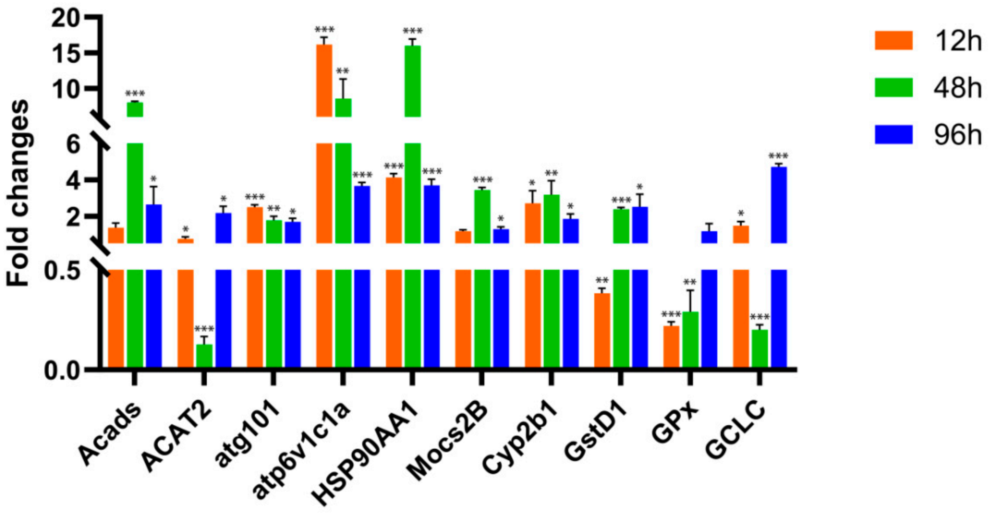

Figure S3. qRT-PCR validation of representative differentially expressed genes (DEGs). The X-axis shows 10 representative DEGs randomly selected from Table 1, which were validated by quantitative real-time PCR (qRT-PCR). The Y-axis represents the fold change, calculated as the relative expression of each gene in the treatment group relative to the control group at each stress time point based on Ct values. In the figure, orange bars represent the fold change results at 12h post-stress, green bars at 48h post-stress, and blue bars at 96h post-stress.Statistical significance was assessed via a two-tailed *t*-test for each stress time point by comparing the treatment group to the control group. Significance levels are annotated as follows: $P < 0.001$ denoted by “***”; $P < 0.01$ denoted by “**”; $P < 0.05$ denoted by “*”.

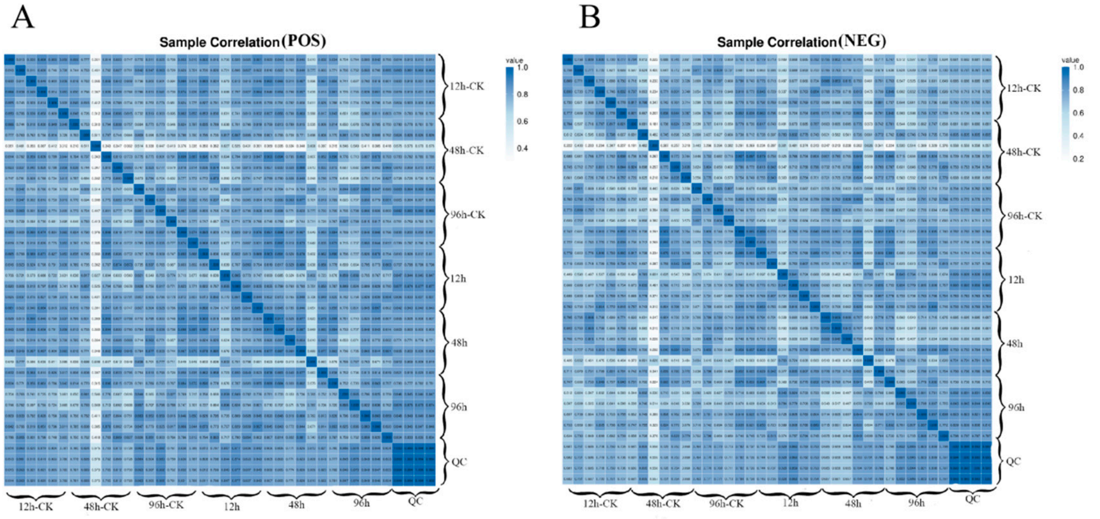

Figure S4. Metabolome Correlation Heatmap. (A) shows the correlation heatmap for the positive ion mode (POS); (B) shows the correlation heatmap for the negative ion mode (NEG). The Y-axis labels 12h, 48h, and 96h represent the experimental group subjected to high ammonia nitrogen stress under low salinity at three time points. The labels 12h-CK, 48h-CK, and 96h-CK denote the control group maintained only in 5‰ salinity aquaculture water without high ammonia nitrogen stress at the same three time points. The expression levels of each pair of samples were extracted to calculate the Pearson correlation coefficient between every two samples. These coefficients were then visually presented in the form of a heatmap to reflect the correlation between any two samples, which allows for the evaluation of the repeatability of intra-group replicate samples.

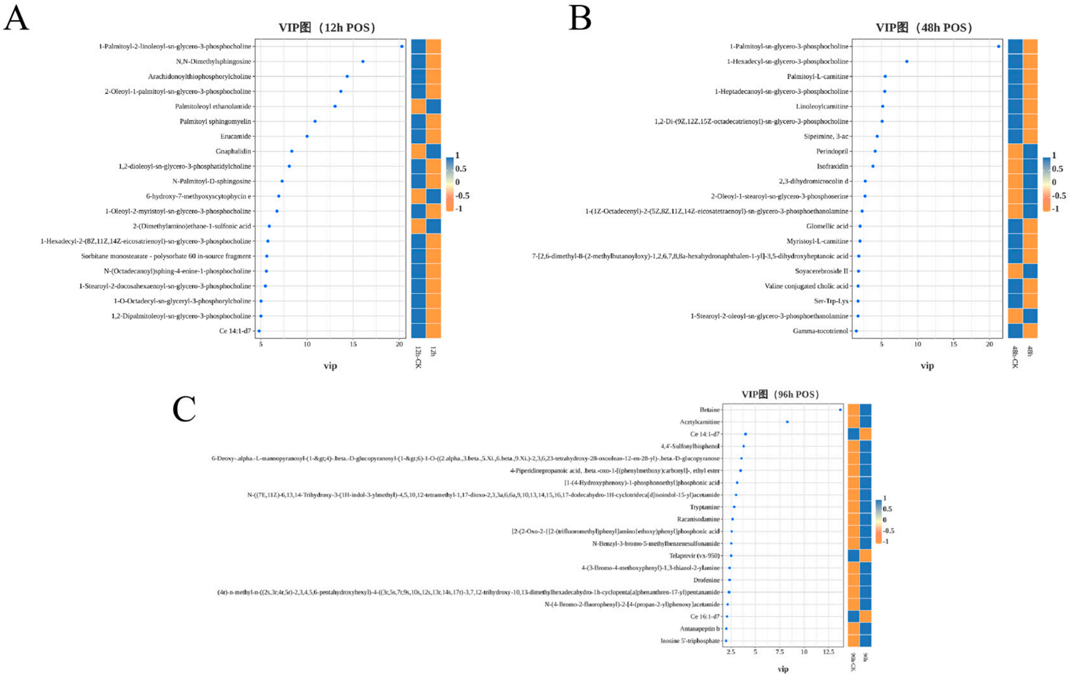

Figure S5. VIP Score Plots of Differentially Expressed Metabolites in Positive Ion. (A) VIP score plot of DEMs in positive ion mode at 12h post-ammonia nitrogen stress; (B) at 48h post-ammonia nitrogen stress; (C) at 96h post-ammonia nitrogen stress.ModeOrthogonal partial least squares discriminant analysis (OPLS-DA) was performed to generate the Variable Importance in Projection (VIP) score for each metabolite. Higher VIP scores reflect a stronger contribution of metabolites to the discrimination between the control and treatment groups. Metabolites with a VIP score > 1 were defined as key differentially expressed metabolites (DEMs), as they exhibit substantial discriminatory power for sample grouping.VIP score plots visually quantify the relative importance of DEMs and their individual contributions to sample differentiation. The X-axis represents VIP scores, while the Y-axis lists the top 20 DEMs ranked by their VIP scores. Metabolic abundances were first normalized by calculating the group mean (average of all biological replicates within each group) and subsequently subjected to z-score transformation for colorimetric visualization. Color coding on the right side denotes the relative abundance changes of metabolites: blueindicates upregulation, and orange indicates downregulation.

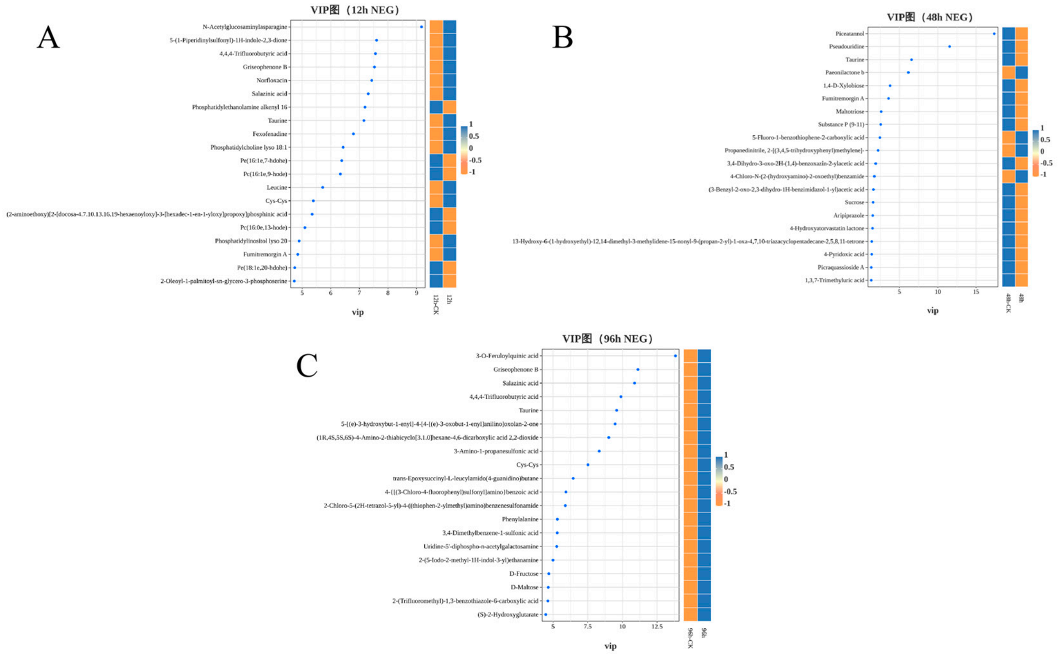

Figure S6. Statistical Plot of VIP Values for Differentially Expressed Metabolites in Negative Ion Mode. (A) VIP plot of differentially expressed metabolites under negative ion mode at 12h post-stress; (B) VIP plot of differentially expressed metabolites under negative ion mode at 48h post-stress; (C) VIP plot of differentially expressed metabolites under negative ion mode at 96h post-stress.Through OPLS-DA analysis, each metabolite yields a VIP value (Variable Importance in Projection). A higher VIP value indicates greater contribution to distinguishing between groups. Metabolites with VIP > 1 exhibit significant differences. The VIP plot illustrates the importance of differentially expressed metabolites and their contribution to sample differentiation. The X-axis displays VIP values, while the Y-axis lists the top 20 differentially expressed metabolites. Metabolic abundance was averaged across samples within each group, followed by z-score

analysis. Colors on the right indicate relative abundance changes: blue denotes upregulation, orange indicates downregulation.
